# Supplementary material for: Informing prevention of stillbirth and preterm birth in Malawi: development of a minimum dataset for health facilities participating in the DIPLOMATIC collaboration
Source: BMJ Open. 2020 Nov 24;10(11):e038859. doi: 10.1136/bmjopen-2020-038859 (PMC7689106; doi:10.1136/bmjopen-2020-038859)
Supplement: Author's manuscript [file bmjopen-2020-038859.draft_revisions.pdf]

BMJ Open is committed to open peer review. As part of this commitment we make the peer review history of every article we publish publicly available.

When an article is published we post the peer reviewers' comments and the authors' responses online. We also post the versions of the paper that were used during peer review. These are the versions that the peer review comments apply to.

The versions of the paper that follow are the versions that were submitted during the peer review process. They are not the versions of record or the final published versions. They should not be cited or distributed as the published version of this manuscript.

BMJ Open is an open access journal and the full, final, typeset and author-corrected version of record of the manuscript is available on our site with no access controls, subscription charges or pay-per-view fees (http://bmjopen.bmj.com).

If you have any questions on BMJ Open's open peer review process please email info.bmjopen@bmj.com

BMJ Open

Informing prevention of stillbirth and preterm birth in Malawi: development of a minimum dataset for health facilities participating in the DIPLOMATIC collaboration

Journal:	*BMJ Open*
Manuscript ID	bmjopen-2020-038859
Article Type:	Original research
Date Submitted by the Author:	26-Mar-2020
Complete List of Authors:	von Wissmann, Beatrix; NHS National Services Scotland, Information Services Division; NHS Greater Glasgow and Clyde, Public Health<br>Wastnedge, Elizabeth; The University of Edinburgh MRC Centre for Reproductive Health<br>Waters, Donald; The University of Edinburgh MRC Centre for Reproductive Health<br>Gadama, Luis; University of Malawi College of Medicine, Department of Obstetrics and Gynaecology<br>Dube, Queen; University of Malawi College of Medicine, Paediatric Department<br>Masesa, Clemens; Malawi-Liverpool-Wellcome Trust Clinical Research Programme<br>Chodzaza, Elizabeth; University of Malawi Kamuzu College of Nursing, Faculty of Midwifery, Neonantal and Reproductive Health Studies<br>Stock, Sarah; The University of Edinburgh Usher Institute of Population Health Sciences and Informatics<br>Reynolds, Rebecca; The University of Edinburgh Centre for Cardiovascular Science, Queen's Medical Research Institute<br>Norrie, John; The University of Edinburgh Usher Institute of Population Health Sciences and Informatics, Edinburgh Clinical Trials Unit<br>Makwakwa, Enita; Malawi Epidemiology and Intervention Research Unit<br>Freyne, Bridget; University of Liverpool Institute of Infection and Global Health; University of Malawi College of Medicine, Paediatric Department<br>Campbell, Harry; The University of Edinburgh Usher Institute of Population Health Sciences and Informatics, Centre for Global Health Research<br>Norman, Jane; University of Bristol, Faculty of Health Sciences<br>Wood, Rachael; NHS National Services Scotland, Information Services Division; University of Edinburgh Salvesen Mindroom Research Centre
Keywords:	Information management < BIOTECHNOLOGY & BIOINFORMATICS, International health services < HEALTH SERVICES ADMINISTRATION & MANAGEMENT, Quality in health care < HEALTH SERVICES ADMINISTRATION & MANAGEMENT, NEONATOLOGY, OBSTETRICS

I, the Submitting Author has the right to grant and does grant on behalf of all authors of the Work (as defined in the below author licence), an exclusive licence and/or a non-exclusive licence for contributions from authors who are: i) UK Crown employees; ii) where BMJ has agreed a CC-BY licence shall apply, and/or iii) in accordance with the terms applicable for US Federal Government officers or employees acting as part of their official duties; on a worldwide, perpetual, irrevocable, royalty-free basis to BMJ Publishing Group Ltd ("BMJ") its licensees and where the relevant Journal is co-owned by BMJ to the co-owners of the Journal, to publish the Work in this journal and any other BMJ products and to exploit all rights, as set out in our licence.

The Submitting Author accepts and understands that any supply made under these terms is made by BMJ to the Submitting Author unless you are acting as an employee on behalf of your employer or a postgraduate student of an affiliated institution which is paying any applicable article publishing charge ("APC") for Open Access articles. Where the Submitting Author wishes to make the Work available on an Open Access basis (and intends to pay the relevant APC), the terms of reuse of such Open Access shall be governed by a Creative Commons licence – details of these licences and which Creative Commons licence will apply to this Work are set out in our licence referred to above.

Other than as permitted in any relevant BMJ Author's Self Archiving Policies, I confirm this Work has not been accepted for publication elsewhere, is not being considered for publication elsewhere and does not duplicate material already published. I confirm all authors consent to publication of this Work and authorise the granting of this licence.

For peer review only

Informing prevention of stillbirth and preterm birth in Malawi: development of a minimum dataset for health facilities participating in the DIPLOMATIC collaboration

Word count: 3354

List of authors:

Beatrix von Wissmann, PhD, NHS National Services Scotland, Information Services Division, Gyle Square, 1 South Gyle Crescent, Edinburgh EH12 9EB, UK, beatrix.vonwissmann@nhs.net, +44 141 2014917 (Corresponding Author)

Elizabeth Wastnedge, MB ChB, MRC Centre for Reproductive Health, The University of Edinburgh, Edinburgh, UK

Donald Waters, MB ChB, MRC Centre for Reproductive Health, The University of Edinburgh, Edinburgh, UK

Luis A Gadama, Department of Obstetrics and Gynaecology, University of Malawi, College of Medicine, Blantyre, Malawi

Queen Dube, Paediatric Department, College of Medicine, University of Malawi, Blantyre, Malawi

Clemens Masesa, Malawi-Liverpool-Wellcome Trust Clinical Research Programme, Blantyre, Malawi

Elizabeth Chodzaza, PhD, RNM, Faculty of Midwifery, Neonantal and Reproductive Health Studies, Kamuzu College of Nursing, University of Malawi, Blantyre, Malawi

Sarah J Stock, PhD, Usher Institute of Population Health Sciences and Informatics, University of Edinburgh, Edinburgh, UK

Rebecca M Reynolds, Prof, PhD, Centre for Cardiovascular Science, University of Edinburgh, Queen's Medical Research Institute, Edinburgh, UK

John Norrie, MSc, Edinburgh Clinical Trials Unit, Usher Institute of Population Health Sciences & Informatics, Edinburgh, UK

Enita Makwakwa, MPH, Malawi Epidemiology and Intervention Research Unit, Lilongwe, Malawi

Bridget Freyne, MB BCh BAO, Institute of Infection & Global Health, University of Liverpool, Liverpool, UK and University of Malawi College of Medicine, Blantyre, Malawi

Harry Campbell, Prof, Centre for Global Health Research, Usher Institute of Population Health Sciences and Informatics, Medical School, University of Edinburgh, Edinburgh, UK

Jane E Norman, Prof, MD, Faculty of Health Sciences, University of Bristol, Bristol, UK

Rachael Wood, PhD, NHS National Services Scotland, Information Services Division, Edinburgh, UK and Salvesen Mindroom Research Centre, University of Edinburgh, Edinburgh, UK

For peer review only - http://bmjopen.bmj.com/site/about/guidelines.xhtml

On behalf of the DIPLOMATIC collaboration (Catherine Bamuya, Esmie Banda, James Boardman, Effie Chipeta, Mia Crampin, Sarah Cunningham Burley, Jean Desire Kabamba, Elizabeth Grant, Caroline Hollins Martin, Aisha Holloway, Khondwhani Kawaza, Doris Kayambo, Corrine Love, Brian Magowan, Monica Malata, Martha Masamba, Fadhila Mazanderani, Gladys Membe- Gadama, Catherine Mkandawire, Patricia Munthali, Peter Mwaba, Shakira Namisengo, Everist Njelesani, Linda Nyondo-Mipando, Hilary Pinnock, Muriel Syacumpi, Frank Taulo, Alexandra Viner)

For peer review only

ABSTRACT

Background

The global research group, DIPLOMATIC (Using eviDence, Implementation science, and a clinical trial PLatform to Optimise MATernal and newborn health in low Income Countries), aims to reduce stillbirths and preterm births and optimise outcomes for babies born preterm. An early objective was to design minimum datasets for routine data collection in healthcare facilities participating in DIPLOMATIC (initially in Malawi), to improve understanding of baseline maternal and neonatal care processes and outcomes, and facilitate the evaluation of improvement interventions and pragmatic clinical trials.

Methods

Published and grey literature was reviewed alongside extensive in-country consultation to define relevant clinical best practice guidance and the existing local data infrastructure and reporting requirements. Identified data item requirements were subject to iterative rounds of consultation with topic experts in Malawi and Scotland, the relevant Malawian professional bodies, and the Ministry of Health in Malawi to ensure relevance, validity, and feasibility.

Results

The resulting minimum datasets cover the maternal and neonatal healthcare journey from antenatal to specialist neonatal care, with provision for effective linkage of records relating to the same mother/baby pair. They can facilitate consistent and precise recording of relevant outcomes (stillbirths, preterm births and neonatal deaths), risk factors and key care processes.

Conclusions

Poor quality routine data on care processes and outcomes constrains healthcare system improvement. The datasets developed for implementation in DIPLOMATIC partner facilities reflect, and hence support delivery of, internationally agreed best practice for maternal and newborn care in low income settings. They have been informed by extensive consultation and are designed to integrate with and strengthen existing structures and processes. Implementation will meet both local reporting requirements and research data needs. This work thus provides a transferable example of strengthening data infrastructure to underpin a learning healthcare system approach in low income settings.

DIPLOMATIC is funded by the UK National Institute for Health Research.

For peer review only

STRENGTH AND LIMITATIONS OF THIS STUDY

- The three interlinking perinatal datasets, developed for the DIPLOMATIC collaboration, collectively cover the maternal and neonatal healthcare journey from antenatal care to neonatal care.
- This integration of data elements along the perinatal care continuum has not previously been implemented in Malawi for the purpose of routine health data collection at primary health centre level.
- The datasets are based on, and can therefore support delivery of, internationally agreed best practice for maternal and newborn care in low income settings.
- The datasets are more extensive than other exemplars of national minimum datasets, but inclusion of additional variables ensures they can integrate with existing systems and meet local data requirements, rather than addressing research requirements only, and also have the capacity to evaluate other Ministry of Health initiatives.
- Iterative rounds of consultation with DIPLOMATIC collaborators and other stakeholders in Malawi were essential to facilitate Malawian ownership, increasing the likelihood that study recommendations can be implemented and scaled up to increase impact.

For peer review only

For peer review only - http://bmjopen.bmj.com/site/about/guidelines.xhtml

INTRODUCTION

The global burden of stillbirths and preterm births remains significant. According to recent global estimates, 2.6 million babies were stillborn at 28 completed weeks gestation or over in 2015.[1] Half of stillborn babies died during labour.[1] Complications of preterm birth (defined as delivery before 37 completed weeks gestation) accounted for nearly one fifth of deaths in under five year old children globally (18%) in 2016.[2] Babies born preterm who survive remain at risk of developmental delay,[3] and long term physical morbidities.[4]

DIPLOMATIC

DIPLOMATIC (Using eviDence, Implementation science, and a clinical trial PLatform to Optimise MATernal and newborn health in low Income Countries) is a National Institute of Health Research (NIHR) funded global research group with partners in Malawi, Zambia and the United Kingdom (UK), which aims to reduce stillbirth and preterm birth, and optimise outcomes for babies born preterm. In Malawi and Zambia, an estimated 14,000 and 13,000 stillbirths occur every year (21.8 and 20.9 stillbirths per 1000 total births respectively).[1] An estimated 68,000 and 76,000 babies per year are born preterm, accounting for 10.5% of births in Malawi and 12% in Zambia.[5] Ranked by increasing rates, Malawi and Zambia were 160th and 155th out of 194 countries for rates of stillbirth in 2015 and 123rd and 127th out of 183 countries for rates of preterm birth in 2014, respectively.[1, 5] DIPLOMATIC aims to develop pragmatic clinical trials to test the effectiveness of evidence based practices and how best to implement them in low income settings. Consistent and accurate data are required from participating healthcare facilities to efficiently monitor intervention implementation and outcomes and hence trial results.

Current perinatal data infrastructure in Malawi

To ensure that the data collection for DIPLOMATIC would integrate with and strengthen existing systems, the collaboration undertook a baseline in depth review of the existing data infrastructure relating to perinatal health in Malawi.

Vital events registration in Malawi is mandated by the National Registration Act of 2010, but coverage of birth and death registration is still incomplete, though efforts to improve coverage are in progress.[6, 7]

Individuals hold their own medical records as ‘health passports’ (structured paper based records), which they present whenever accessing healthcare. For women of childbearing age, the health passport records a summary of medical and obstetric history, contraceptives, antenatal care (ANC),

For peer review only - http://bmjopen.bmj.com/site/about/guidelines.xhtml

and the delivery record. Neonates receive their own passport at birth. Most district and central hospitals maintain paper based patient notes when providing inpatient perinatal care. For specialist neonatal care, a standardised admission form is available. A brief note is made in the patients' health passport at discharge,[8] which is usually the main source of information to clinicians on patients' history in case of readmission, as retrieval of patient notes held by healthcare facilities is difficult. Neonatal and maternal records are not routinely linked, and if the mother is not present or deceased, her information is not available for the care of the neonate. An electronic patient record system for ANC is available, but uptake is limited to date (24 out of 85 hospitals and 12 out of 542 health centres across Malawi).[9] An application for immediate electronic data capture on neonatal admissions (NeoTree) was developed recently and tested in one hospital.[10] This application is designed as a decision support tool, to improve quality of care, and does not have any reporting functionality for integration with local data systems as yet.[10]

In addition to individual patient notes, healthcare facilities maintain 'registers' (individual level paper logs) for clinical care including ANC, maternity/birth record, neonatal resuscitation, and Kangaroo Mother Care (KMC), with partial overlap in data items recorded. Aggregate data on healthcare activity are regularly extracted from registers and reported to the Ministry of Health (MoH), via the District Health Information System.[8]

Importance of high quality data

Stillbirths, and morbidity and mortality associated with preterm births are markers of maternal health and markers of access to high quality care in pregnancy, especially around the time of childbirth.[11, 12] However, global and national estimates of stillbirths and premature births are frequently constrained by data quality.[1, 5, 13] High quality data are required to measure the burden of stillbirths and premature births, to assess effectiveness of interventions, and to monitor the benefits of investment to implement evidence based interventions (or the adverse consequences of a lack of investment). The importance of improving medical records and healthcare information systems, in order to allow monitoring and evaluation, and to improve performance and ultimately outcomes, has also been highlighted as the second of eight WHO standards for improving the quality of maternal and newborn care in health facilities.[14]

Aim of this work

The aim of this work was to design a minimum dataset, which can be piloted and implemented across all healthcare facilities participating in DIPLOMATIC, to enable electronic collection of consistent and accurate data on perinatal outcomes, risk factors and the coverage of key care

For peer review only - http://bmjopen.bmj.com/site/about/guidelines.xhtml

processes and treatments. Whilst the DIPLOMATIC collaboration spans both Zambia and Malawi, this initial work focused on Malawi, with a view to transferring the equivalent data collection, adapted to the Zambian context, in a second stage.

METHODS

The minimum datasets were drawn up using a multimodal approach, building on review of the literature and existing data infrastructure for identification of data requirements, followed by consultation with project collaborators and wider local stakeholders in Malawi.[15, 16]

Identification of data requirements

Health passports, neonatal admission forms, and standard registers and the corresponding aggregate data returns to the MoH were reviewed. Variables required for the standard registers and to fulfil aggregate reporting requirements to the MoH were identified for inclusion in the datasets. The resulting list of variables was compared to relevant WHO guidance on ANC,[17] maternal health ,[18] and newborn health,[19] to ensure that the proposed datasets could be used to assess coverage of recommended clinical best practice. Finally, agreed quality indicators for maternity and neonatal services,[14, 20, 21] and existing minimum datasets,[22, 23] were reviewed to identify additional relevant variables. Wherever possible, international norms and standards were used to underpin definitions.[24]

Consultation

Identification of the data requirements was followed by iterative rounds of consultation to refine the datasets based on critical evaluation by DIPLOMATIC collaborators and wider stakeholders in Malawi, who provided topic expertise and insights on Malawian health data structure and management:

DIPLOMATIC collaborators were consulted on the appropriateness and feasibility of the draft datasets. In addition to requesting electronic feedback from all collaborators, phone, skype and e-mail conversations with relevant Malawi based experts amongst the collaborators ensured detailed input from obstetricians, paediatricians, midwives, epidemiologists and data scientists. Feedback was recorded against the relevant variables. Where conflicting comments were received, alignment with existing data infrastructure was prioritised, to ensure the data would meet local requirements. The datasets were updated accordingly and shared again with collaborators and discussed at the DIPLOMATIC management group. The draft datasets were shared with relevant professional bodies

For peer review only - http://bmjopen.bmj.com/site/about/guidelines.xhtml

in Malawi (Nurses and Midwives Council, Association of Obstetricians and Gynaecologists, Association of Paediatricians) facilitated by DIPLOMATIC collaborators who are members of the respective bodies. The draft datasets were also shared with colleagues from the MoH Directorate of Reproductive Health and Directorate of Quality Management and Digital Strategy. Finally, the datasets were presented to the Safe Motherhood Technical Working Group, for endorsement by the MoH. The Technical Working Group has multi-sectoral membership including representation from the MoH and the health sector as well as the third sector and academia, and acts as a platform for policy dialogue and coordination of work.

For peer review only

Patient and Public involvement

Patients were not involved in the development of the datasets, but as set out in the DIPLOMATIC stakeholder engagement plan, the extensive experience of local host institutions for the DIPLOMATIC project (University of Malawi College of Medicine; Malawi Liverpool Wellcome Trust, Malawi Epidemiology and Intervention Research Unit) will be used to develop a program for public sensitisation around the interventions to be implemented (and to be evaluated using the datasets as described here), which will be supported by the Science Communication team at Malawi Liverpool Wellcome Trust. This will involve consultation with Community Groups, Women's Groups, Patients and Healthcare Workers and will involve multimedia platforms and local meetings.

RESULTS

Minimum dataset

Three minimum datasets were drafted to cover ANC, inpatient maternity care (including delivery and care of neonates in maternity settings, and care for miscarriages or complications of miscarriages), and specialist neonatal care. Personal identifiers and demographic variables were included in each of the three datasets to ensure individuals can be uniquely identified and their records linked for care across different settings and at different facilities (horizontal linkage for the same individual during a single pregnancy and subsequent pregnancies, and vertical linkage for mothers and their babies). To mitigate the risk of incomplete information due to patients receiving some elements of their care in facilities not participating in DIPLOMATIC, the datasets were designed for a hybrid approach between contemporaneous and retrospective data collection. Data would ideally be collected contemporaneously in each of the settings (ANC and maternity and neonatal inpatient) as care is completed. However, there is the option to capture retrospective summary information on

For peer review only - http://bmjopen.bmj.com/site/about/guidelines.xhtml

ANC within the maternity dataset, and on maternity/delivery care within the neonatal care dataset, if required (Figure 1).

Figure 1: DIPLOMATIC datasets structure [submitted as separate file]

The three datasets (summarised in Tables 1-3, available in full on Open Science Framework[15]) included variables to record:

- the main outcomes of interest to the collaboration (stillbirths, preterm births and neonatal deaths), including variables to record estimated date of delivery (EDD), and pregnancy outcome, and ICD10 coding for cause of death for stillbirths and neonatal deaths where available.
- key clinical data on potential risk factors and mediators for these outcomes (factors on the causal pathway), including the required variables on obstetric history to derive the Robson criteria (which classify women undergoing caesarean sections into groups defined by a global standard, for comparison of caesarean rates between and within healthcare facilities), underlying maternal conditions of relevance to the current pregnancy, and neonatal observations and underlying conditions.
- process measures for key aspects of routine healthcare as potential modifiers for these outcomes (factors increasing/ reducing risk of adverse outcomes), for ANC, delivery and immediate care of the newborn (including emergency interventions), and specialist neonatal care.

For peer review only

For peer review only - http://bmjopen.bmj.com/site/about/guidelines.xhtml

Table 1: Antenatal care (ANC) dataset structure and summary of included variables

when recorded	group	variable	comment
Booking contact (cross check ID at each contact)	unique ID	maternal unique ID	record linkage
	ID& demography	maternal names (incl parents' names), date of birth, district & neighbourhood<br>maternal education<br>maternal local ID & local file numbers	record linkage failsafe (building on experience of requirements to uniquely identify individuals from Karonga DHSS*)<br>measure of socioeconomic status<br>cross-reference local files
All ANC contacts	ANC episode details	facility, level of ANC and care provider	level of care
	contact date	contact date	
	observations & tests	fetal heart rate, symphysis fundal height, any ultrasound scan (USS), maternal haemoglobin, blood pressure, urine protein, infections (syphilis, HIV and Hep B status)	for any USS after booking reason is recorded, many of these observations & tests are required for mandatory reporting to MoH
	care & treatment	tetanus toxoid vaccine doses, malaria prophylaxis, iron and folic acid tablets, preventive anthelmintic,<br>For HIV positive women: drugs for prevention of mother to child transmission (PMTCT)	many of these process measures are required for mandatory reporting to MoH
	high risk pregnancy indicator	indicator of whether woman meets any of the WHO high risk criteria	
Booking contact	EDD (and method of estimation)	EDD; USS before or at booking; LMP	EDD is recorded at booking alongside methods used for the estimate (USS, LMP, symphysis fundal height), EDD can subsequently only be altered once - and only on the basis of additional information provided by a first USS occuring after the booking visit
	obstetric, medical & behavioural history	obstetric history (previous pregnancies, deliveries and c-sections)<br>medical history (weight, height, blood group, HIV status, tetanus vaccination history)<br>behaviour (smoking, alcohol, drugs)	allows derivation of Robson criteria

*Karonga Health and Demographic Health Surveillance System (HDSS)[25]

For peer review only - http://bmjopen.bmj.com/site/about/guidelines.xhtml

Abbreviations used in table 1: Antenatal care (ANC), caesarean section (c-section), estimated date of delivery (EDD), human immunodeficiency virus (HIV), Hepatitis B (Hep B), identifier (ID), International Classification of Diseases version 10 (ICD10), last menstrual period (LMP), Ministry of Health (MoH), prevention of mother to child transmission (PMTCT), ultrasound scan (USS), World Health Organization (WHO)

For peer review only

For peer review only - http://bmjopen.bmj.com/site/about/guidelines.xhtml

Table 2: Maternal discharge dataset (including delivery record) structure and summary of included variables

when recorded	group	variable	comments
All discharges	maternal unique ID	maternal unique ID	record linkage
	ID& demography	maternal names (incl parents' names), date of birth, district & neighbourhood	record linkage failsafe (building on experience of requirements to uniquely identify individuals from Karonga HDSS[1])
		maternal education	measure of socioeconomic status
		maternal local ID & local file numbers	cross-reference local files
All discharges	episode dates & details	admission and discharge dates, facility, admission from, discharge to<br>delivery and miscarriage episodes only: care provider staff group	
	condition & outcome	reason for admission (category and cause coded in ICD10), condition on discharge	condition on discharge indicates the outcome i.e. miscarriage, delivered or is still pregnant at the end of the care episode
Delivery and miscarriage episodes	ANC summary	obstetric, medical & behavioural history summary	to be autofilled if ANC took place in DIPLOMATIC participating facility, to be manually recorded retrospectively if ANC took place in facility not participating in DIPLOMATIC
		date of booking contact	
		care & treatment summary	
		high risk pregnancy indicator, multiple pregnancy	
		EDD (and methods of estimation) summary	
Delivery record mother	outcome & details	number of babies, outcome of pregnancy, birth order, date, time, place, presentation, mode, indications for assisted delivery,	
	observation & tests	partograph used; HIV test, duration of stages of labour,	many of these observations & tests are required for mandatory reporting to MoH
	care & treatments	<u>preventive / routine:</u> antenatal steroids, magnesium sulfate, labour induction, augmentation of labour, analgesia, uterotonic for active management third stage of labour, episiotomy, companion present;<br><u>emergency:</u> uterotonic, anticonvulsive, antibiotic, blood transfusion, manual removal of placenta	many of these process measures are required for mandatory reporting to MoH

For peer review only - http://bmjopen.bmj.com/site/about/guidelines.xhtml

	discharge record	iron and folic acid tablets, preventive anthelminitic, contraception	
Delivery record baby	unique ID	baby unique ID	
	observations	all: sex, weight; livebirths: Apgar score, crown-heel length, head circumference	
	care & treatment	routine: dried & wrapped, delayed cord clamping, cord cut with sterile blade, skin to skin contact initiated, breastfeeding initiated, Vitamin K given, tetracycline eye ointment, BCG & OPV vaccine, PMTCT initiated (if mother HIV positive); emergency/supportive: resuscitation at birth, systemic antibiotics, kangaroo mother care (KMC) details, other thermal support	many of these process measures are required for mandatory reporting to MoH
	condition	congenital malformations, other perinatal complications	
	discharge record	date, feeding at discharge (mode & content), on KMC at discharge, discharge provider	
Miscarriages	miscarriage type & management	miscarriage type & management	
Stillbirths & Neonatal deaths	cause of death	immediate & underlying cause of death (coded in ICD10)	

*Karonga Health and Demographic Health Surveillance System (HDSS)[25]

Abbreviations used in table 2: Antenatal care (ANC), Bacillus Calmette–Guérin tuberculosis vaccine (BCG), identifier (ID), estimated date of delivery (EDD), International Classification of Diseases version 10 (ICD10), kangaroo mother care (KMC), Ministry of Health (MoH), Oral Polio Vaccine (OPV), prevention of mother to child transmission (PMTCT)

For peer review only - http://bmjopen.bmj.com/site/about/guidelines.xhtml

Table 3: Specialist neonatal care dataset structure and summary of included variables

when recorded	group	variable	use
All discharges	baby unique ID	baby unique ID	record linkage
	baby ID& demography	baby names, date of birth, sex, district & neighbourhood	record linkage failsafe
		baby local ID & local file numbers	cross-reference local files
	maternal unique ID	maternal unique ID	record linkage
	maternal ID& demography	maternal names (incl parents' names), date of birth, district & neighbourhood	record linkage failsafe (building on experience of requirements to uniquely identify individuals from Karonga HDSS[1])
		maternal education	measure of socioeconomic status
		maternal local ID & local file numbers	cross-reference local files
All discharges	episode dates & details	admission and discharge dates, facility, admission from, discharge to	
	condition	reason for admission (category and cause coded in ICD10),	
	observations	temperature & weight at admission	
	care & treatment	emergency/supportive: major invasive or therapeutic procedures, kangaroo mother care (KMC) details, other thermal support, respiratory support	many of these observations & tests are required for mandatory reporting to MoH
	discharge record	date, weight at discharge, feeding at discharge (mode & content), on KMC at discharge, discharge provider	
All discharges	Delivery record mother summary	final EDD	to be autofilled if delivery took place in DIPLOMATIC participating facility, to be manually recorded retrospectively if delivery took place in facility not participating in DIPLOMATIC
		outcome & details	
		observation & tests	
		care & treatments	
	Delivery record baby summary	observations	
		care & treatments	
Neonatal deaths	cause of death	immediate & underlying cause of death (coded in ICD10)	

For peer review only - http://bmjopen.bmj.com/site/about/guidelines.xhtml

*Karonga Health and Demographic Health Surveillance System (HDSS)[25]

Abbreviations used in table 3: Antenatal care (ANC), identifier (ID), estimated date of delivery (EDD), International Classification of Diseases version 10 (ICD10), kangaroo mother care (KMC), Ministry of Health (MoH), prevention of mother to child transmission (PMTCT)

For peer review only

For peer review only - http://bmjopen.bmj.com/site/about/guidelines.xhtml

All variables and response options were supported by clear definitions, using internationally recognised norms where possible.[24). Validation cross-checks at data entry were built in to support data quality by design. The datasets were designed to include all required variables to replicate the standard registers used in the respective settings [ANC , maternity, helping babies breathe (neonatal resuscitation), KMC] and to allow generation of the respective mandatory reports for the MoH via the District Health Information System .

Consultation with the DIPLOMATIC collaborators, and wider stakeholders and professional bodies in Malawi resulted in refinement of a number of variables, including additional personal identifiers to create a failsafe for record linkage (based on local experience[25]), and the combination of variables that allow recording of the EDD and the method of estimation. Consultation input also ensured that the response options for all process measures were appropriate for the Malawian healthcare context, including response options to record that items were out of stock. The datasets were endorsed by the MoH through approval by the Safe Motherhood Technical Working Group.

DISCUSSION

The DIPLOMATIC minimum datasets were designed to cover the maternal and neonatal healthcare journey from ANC to specialist neonatal care. The datasets encompass the required elements to allow precise recording of the relevant outcomes (stillbirths, preterm births and neonatal deaths), as well as risk factors and key care processes. The datasets are aligned with international standards and definitions to ensure comparability of the results, and will enable DIPLOMATIC and the MoH to evaluate implementation and effectiveness of interventions (including care in line with existing policies). They were designed to integrate with and strengthen local data systems and meet reporting requirements, and to meet data needs to support decision making and learning for quality improvement in line with the WHO standards for improving the quality of maternal and newborn care in health facilities.[14] Consultation and refinement will need to continue during pilots and implementation, to ensure the datasets remain aligned with MoH reporting requirements and to enable synergies with projects in the fast moving field of EMR developments.

Data collection on preterm births and stillbirths is reliant on accurate information on gestation, which underpins the definitions of both concepts. WHO recommends the integration of an ultrasound scan before 24 weeks of gestation into routine ANC, as the most accurate method of determining gestation.[17] At present, ultrasound scans are not part of routine ANC in Malawi, but

For peer review only - http://bmjopen.bmj.com/site/about/guidelines.xhtml

this was identified as a key evidence-based priority and one of the interventions to be implemented in the healthcare facilities participating in DIPLOMATIC.

The methods of assessment of gestational age not only determine the precision of estimates for individual pregnancies, but can also lead to systematic differences in population level estimates of prematurity rates,[5] (as exemplified by changes in reporting in the United States [26]). However, most data sources contributing to recent global, regional and national estimates of premature birth rates reported insufficient details on the methods to determine gestational age, to allow the estimates to take this parameter into account.[5] In contrast, the DIPLOMATIC datasets include the relevant parameters to allow recording of whether and when an ultrasound scan was carried out to date the pregnancy, or which other method were used (last menstrual period, symphysis fundal height), alone or in combination to estimate gestation. These datasets thus allow estimates of preterm birth rates to be adjusted for the methods used to estimate gestational age. The datasets will also allow other important factors to be taken into account, such as multiplicity, and onset of delivery (spontaneous or provider initiated) for analysing both live and total births,[13] and maternal risk factors such as body mass index (BMI) and hypertensive conditions.[27]

The datasets include process measures on different elements of routine care. These elements serve to meet local reporting requirements, and by recording uptake of evidence based antenatal, maternal and neonatal care as recommended by WHO,[17-19] they facilitate monitoring of improvements in quality of care. Malawi has adopted the 'Every Newborn Action Plan',[20, 28] and is one of the countries participating in the 'Network for Improving Quality of Care for Maternal, Newborn and Child Health', which aims to halve newborn and maternal deaths, and intrapartum stillbirths in participating countries over five years.[29] The process measures which will be captured in the DIPLOMATIC datasets are closely aligned to the WHO standards for improving quality of maternal and newborn care in healthcare settings,[14] and will facilitate reporting against a large proportion of the stipulated quality measures.

Supporting staff to facilitate optimal use of the data will be an important part of piloting and implementation of the datasets. Electronic data can significantly reduce the work required to extract data for audits and patient reviews (compared to extraction from paper records). Experiencing benefits from the data will enhance ownership and buy in from clinical staff, and will be a prerequisite to ensuring sustainability of the datasets. High quality data providing continuous feedback on quality and safety of care and patient outcomes to healthcare providers can underpin a 'learning health system', which allows data intelligence to foster learning and inform decisions.[30] Data linkage plays an important part in this when transfer of patients across facilities is common.

For peer review only - http://bmjopen.bmj.com/site/about/guidelines.xhtml

Learning health system approaches have been shown to result in improved data and care quality in limited-resource settings similar to Malawi.[31] The DIPLOMATIC datasets have the potential to facilitate a learning health system approach.

Strengths and limitations

In line with recommended practice, the datasets were designed using a multimodal approach, combining a systematic review of the literature and contextual information with expert input.[32] Delphi methods to gain expert consensus were considered, but deemed unsuitable due to the large number of variables, many of which form interconnected constructs and could thus not be changed independently of each other. Instead of a Delphi survey, iterative rounds of consultation with DIPLOMATIC collaborators and other stakeholders in Malawi were undertaken. Group discussions were helpful to find agreement on complex constructs, e.g. recording of EDD and methods used for its estimation. Whilst this process also took considerable time to complete, partly due to competing demands for the time of all of the relevant experts, it facilitated agreement on the variables to be prioritised for inclusion in the datasets. Sharing the draft datasets with professional associations and the relevant directorates of the MoH Malawi, and gaining approval by the Safe Motherhood Technical Working Group, was important to ensure there were no objections to the datasets, and to strengthen Malawian ownership, increasing the likelihood that any recommendations arising from future study findings can be implemented and scaled up to increase impact.

The DIPLOMATIC datasets are more extensive than other exemplars of national minimum datasets on maternal and neonatal health.[22, 23] This poses a challenge for implementation of these datasets in the context of stretched clinical staff, a background of predominantly paper based data recording and mixed success of electronic data systems.[8] However it was deemed essential to ensure that the datasets incorporate all data items required for standard registers and can fulfil the Malawian national reporting obligations to the MoH, so the datasets can be embedded in existing systems and strengthen these (in line with the 'record once, use often' principle). This approach avoided creation of parallel datasets specific only to the research data needs, which would have been smaller, but would have overlapped to a large extent with existing paper based registers, leading to duplication of effort and no benefit to the existing system.

CONCLUSIONS

By combining contextual work with expert input, it was possible to design datasets, which build on existing structures, and can support local reporting requirements, as well as meeting the research

For peer review only - http://bmjopen.bmj.com/site/about/guidelines.xhtml

data needs of DIPLOMATIC. The work to design the datasets highlights the need for a collaborative approach to ensure synergies and to strengthen existing systems, and avoid duplication of data and effort. Consultation on data collection is also essential to ensure local ownership of the data and any findings arising from it. The datasets have the potential to underpin a 'learning health system' approach, and their piloting and implementation will be aimed at supporting quality of care to improve outcomes. Implementation of these datasets could thus contribute to fulfilling the aims of the Network for Improving Quality of Care for Maternal, Newborn and Child Health, in particular to strengthening of the health information system, 'to enable early, appropriate action to improve the care of every woman and newborn' (Standard 2).[14] The datasets may also serve beyond the use by facilities participating in DIPLOMATIC, most immediately as an exemplar on how to incorporate methods of gestation estimates into routine data collection, in settings where access to early pregnancy ultrasound is limited and different methods are likely to be in use. In a wider context they also serve as a transferable example of strengthening data infrastructure and use of robust data, to underpin a learning healthcare system approach in low income settings.

DATA SHARING STATEMENT

The full specifications of the datasets, the development of which is described in this manuscript, are available on Open Science Framework: von Wissmann B, Gadama L, Dube Q, et al. The DIPLOMATIC Collaboration, Work Package 2: Core datasets. 2020. https://osf.io/d3s7j [15].

ACKNOWLEDGEMENTS

This work would not have been possible without consultation with stakeholders from the Ministry of Health Malawi, the Malawian Nurses and Midwives Council, the Malawian Association of Obstetricians and Gynaecologists, and the Malawian Association of Paediatricians.

We would like to thank Jakub Adamów for producing the graphic for this paper.

CONTRIBUTORS

BvW and RW designed the protocol for the development of the datasets and drafted the first version of the datasets. BvW wrote the first draft of the manuscript. BvW, EW, DW and RW synthesised the information needed to identify the dataset requirements. LAG, QD, CM, EC provided expert input on the dataset requirements, and current data infrastructure and management in Malawi. LAG, QD and EC led on engagement with the professional bodies. All authors provided intellectual input to developing the datasets. All authors provided input to drafting the manuscript.

For peer review only

For peer review only - http://bmjopen.bmj.com/site/about/guidelines.xhtml

COMPETING INTERESTS

SJS reports grants from NIHR HTA, non-financial support from HOLOGIC, non-financial support from PARSAGEN, non-financial support from MEDIX BIOCHEMICA, during the conduct of the study; and SJS declares being a member of the HTA General committee.

JN reports membership of the following NIHR boards: Commissioning Priority Review Decision-making committee (2015); Health Technology Assessment (HTA) Commissioning Board (2010-2016); HTA Commissioning Sub-Board (Expression of Interest) (2014); HTA Funding Boards Policy Group (2016-2019); HTA General Board (2016-2019); HTA Post-Board funding teleconference (2016-2019); NIHR Clinical Trials Unit Standing Advisory Committee (2018-present); NIHR HTA and Efficacy and Mechanism Evaluation Editorial Board (2014-2019); Pre-exposure Prophylaxis Impact Review Panel (2017).

JEN reports being named as Principal Investigator on government and charitable research grants to her institution which aim to improve pregnancy outcome. In the last three years she has provided consultancy to Pharma companies GSK and Dilafor: her institution was remunerated for this. Her institution has received travel and subsistence expenses from Merck to facilitate her speaking at a Merck sponsored symposium on metformin. She is on Subpanel A1 for REF, and on a Wellcome Trust Science interview panel, and receive personal renumeration for each.

FUNDING

This research was commissioned by the National Institute for Health Research (NIHR) (GHR Project: 17/63/08 DIPLOMATIC collaboration) using Official Development Assistance funding. The views expressed in this publication are those of the authors and not necessarily those of the NIHR or the Department of Health and Social Care.

SJS is funded by a Wellcome Trust Clinical Career Development Fellowship (209560).

For peer review only - http://bmjopen.bmj.com/site/about/guidelines.xhtml

REFERENCES

1. Blencowe H, Cousens S, Jassir FB, et al. National, regional, and worldwide estimates of stillbirth rates in 2015, with trends from 2000: a systematic analysis. Lancet Glob Health. 2016;4(2):e98-e108. doi: 10.1016/S2214-109X(15)00275-2.

2. UN Inter-Agency Group for Child Mortality Estimation. Levels and trends in child mortality: Report 2017. New York; 2017. https://www.un.org/en/development/desa/population/publications/mortality/child-mortality-report-2017.asp (accessed 26/10/2019)

3. Bhutta AT, Cleves MA, Casey PH, et al. Cognitive and behavioral outcomes of school-aged children who were born preterm: a meta-analysis. JAMA. 2002;288(6):728-37.

4. Moster D, Lie RT, Markestad T. Long-term medical and social consequences of preterm birth. N Engl J Med. 2008;359(3):262-73. doi: 10.1056/NEJMoa0706475.

5. Chawanpaiboon S, Vogel JP, Moller AB, et al. Global, regional, and national estimates of levels of preterm birth in 2014: a systematic review and modelling analysis. Lancet Glob Health. 2019;7(1):e37-e46. doi: 10.1016/S2214-109X(18)30451-0.

6. Gadabu OJ, Ben-Smith A, Douglas GP, et al. Scaling up electronic village registers for measuring vital statistics in rural villages in Malawi. Public Health Action. 2018;8(2):79-84. doi: 10.5588/pha.17.0116.

7. Nichols EK, Giles D, Kang'oma S, et al. Rapid assessment of Malawi's civil registration and vital statistics system. Public health action. 2015;5(3):162-4. doi: 10.5588/pha.15.0021.

8. Tough AG, Lihoma P. Medical record keeping systems in Malawi: Is there a case for hybrid systems and intermediate technologies? Records Management Journal. 2018;28(3):265-77. doi.org/10.1108/RMJ-02-2018-0004

9. Baobab Health Trust. Where we work - Electronic medical record systems deployed in various health facilities nationwide. 2019 [27/05/2019]. http://baobabhealth.org/wherewework.php (accessed 27/05/2019).

10. Crehan C, Kesler E, Nambiar B, et al. The NeoTree application: developing an integrated mHealth solution to improve quality of newborn care and survival in a district hospital in Malawi. BMJ Glob Health. 2019;4(1):e000860. doi: 10.1136/bmjgh-2018-000860

11. Graham W, Woodd S, Byass P, et al. Diversity and divergence: the dynamic burden of poor maternal health. Lancet. 2016;388(10056):2164-75. doi: 10.1016/S0140-6736(16)31533-1. Epub 2016 Sep 16.

12. Lawn JE, Blencowe H, Waiswa P, et al. Stillbirths: rates, risk factors, and acceleration towards 2030. Lancet. 2016;387(10018):587-603. doi: 10.1016/S0140-6736(15)00837-5. Epub 2016 Jan 19.

13. Lee AC, Blencowe H, Lawn JE. Small babies, big numbers: global estimates of preterm birth. Lancet Glob Health. 2019;7(1):e2-e3. doi: 10.1016/S2214-109X(18)30484-4. Epub 2018 Oct 30.

14. World Health Organization. Standards for improving the quality of maternal and newborn care in health facilities. Geneva: WHO; 2016. https://www.who.int/maternal_child_adolescent/documents/improving-maternal-newborn-care-quality/en/ (accessed 26/10/2019).

15. von Wissmann B, Gadama L, Dube Q, et al. The DIPLOMATIC Collaboration, Work Package 2: Core datasets. 2020. https://osf.io/d3s7j (accessed 24/02/2020).

For peer review only - http://bmjopen.bmj.com/site/about/guidelines.xhtml

16. von Wissmann B, Wastnedge E, Waters D, et al. The DIPLOMATIC Collaboration, Work package 2: Contextual information. 2020. https://osf.io/dxwgy (accessed 24/02/2020).

17. World Health Organization. WHO recommendations on antenatal care for a positive pregnancy experience. Geneva: WHO; 2016. https://www.who.int/reproductivehealth/publications/maternal_perinatal_health/anc-positive-pregnancy-experience/en/ (accessed 26/10/2019).

18. World Health Organization. WHO Recommendations on maternal health. Geneva: WHO; 2017. https://www.who.int/maternal_child_adolescent/documents/maternal-health-recommendations/en/ (accessed 26/10/2019).

19. World Health Organization. WHO Recommendations on newborn health. Geneva: WHO; 2017. https://www.who.int/maternal_child_adolescent/documents/newborn-health-recommendations/en/ (accessed 26/10/2019).

20. World Health Organization. Every Newborn: an action plan to end preventable deaths. Geneva; 2014. https://www.who.int/maternal_child_adolescent/newborns/every-newborn/en/ (accessed 26/10/2019).

21. COMET initiative. Core Outcomes in Neonatology (COIN). 2018. http://www.comet-initiative.org/studies/details/842 (accessed 26/10/2019)

22. National Services Scotland Information Services Division. Scottish Morbidity Records 02 (SMR02) Maternity Inpatient and Day Case. http://www.ndc.scot.nhs.uk/Data-Dictionary/SMR-Datasets/SMR02-Maternity-Inpatient-and-Day-Case/ (accessed 26/10/2019).

23. National Services Scotland Information Services Division. Scottish Birth Records. http://www.ndc.scot.nhs.uk/National-Datasets/data.asp?SubID=2 (accessed 26/10/2019).

24. World Health Organization. WHO: recommended definitions, terminology and format for statistical tables related to the perinatal period and use of a new certificate for cause of perinatal deaths. Modifications recommended by FIGO as amended October 14, 1976. Acta Obstet Gynecol Scand. 1977;56(3):247-53.

25. Crampin AC, Dube A, Mboma S, Price, et al. Profile: the Karonga Health and Demographic Surveillance System. International journal of epidemiology. 2012;41(3):676-85. doi: 10.1093/ije/dys088. Epub 2012 Jun 22.

26. Martin JA, Osterman MJK, Kirmeyer SE, et al.. Measuring gestational age in vital statistics data: transitioning to the obstetric estimate. National Vital Statistics Reports. 2015;64(5).

27. Goldenberg RL, Culhane JF, Iams JD, et al. Epidemiology and causes of preterm birth. Lancet. 2008;371(9606):75-84. doi: 10.1016/S0140-6736(08)60074-4.

28. Malawi Ministry of Health. Every Newborn Action Plan: An action plan to end preventable neonatal deaths in Malawi. Lilongwe: Malawi Ministry of Health; 2015. https://www.who.int/pmnch/media/events/2015/malawi_enap.pdf (accessed 26/10/2019).

29. World Health Organization. Quality, Equity, Dignity. A Network for Improving Quality of Care for Maternal, Newborn and Child Health. Geneva: WHO; 2017. https://www.who.int/maternal_child_adolescent/topics/quality-of-care/quality-of-care-brief-qed.pdf?ua=1 (accessed 26/10/2019).

30. Budrionis A, Bellika JG. The Learning Healthcare System: Where are we now? A systematic review. J Biomed Inform. 2016;64:87-92. doi: 10.1016/j.jbi.2016.09.018. Epub 2016 Sep 28.

For peer review only - http://bmjopen.bmj.com/site/about/guidelines.xhtml

31. Tuti T, Bitok M, Malla L, et al. Improving documentation of clinical care within a clinical information network: an essential initial step in efforts to understand and improve care in Kenyan hospitals. BMJ Glob Health. 2016;1(1):e000028.

32. Svensson-Ranallo PA, Adam TJ, Sainfort F. A framework and standardized methodology for developing minimum clinical datasets. AMIA Jt Summits Transl Sci Proc. 2011;2011:54-8.

For peer review only

For peer review only - http://bmjopen.bmj.com/site/about/guidelines.xhtml

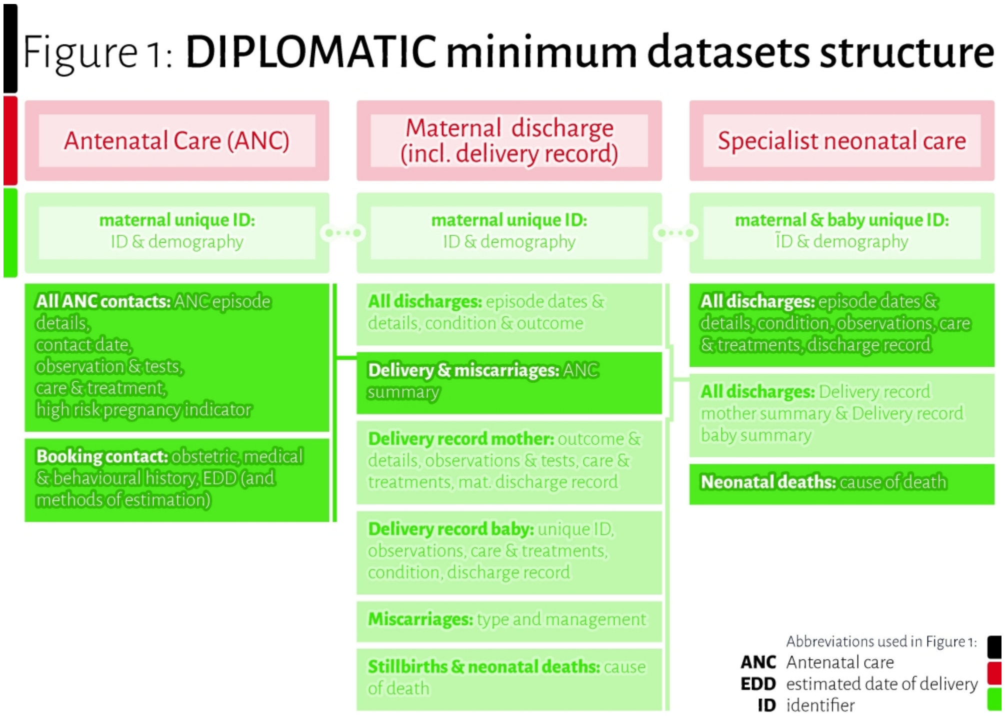

180x128mm (600 x 600 DPI)

BMJ Open

Informing prevention of stillbirth and preterm birth in Malawi: development of a minimum dataset for health facilities participating in the DIPLOMATIC collaboration

Journal:	*BMJ Open*
Manuscript ID	bmjopen-2020-038859.R1
Article Type:	Original research
Date Submitted by the Author:	27-Jul-2020
Complete List of Authors:	von Wissmann, Beatrix; NHS National Services Scotland, Information Services Division; NHS Greater Glasgow and Clyde, Public Health<br>Wastnedge, Elizabeth; The University of Edinburgh MRC Centre for Reproductive Health<br>Waters, Donald; The University of Edinburgh MRC Centre for Reproductive Health<br>Gadama, Luis; University of Malawi College of Medicine, Department of Obstetrics and Gynaecology<br>Dube, Queen; University of Malawi College of Medicine, Paediatric Department<br>Masesa, Clemens; Malawi-Liverpool-Wellcome Trust Clinical Research Programme<br>Chodzaza, Elizabeth; University of Malawi Kamuzu College of Nursing, Faculty of Midwifery, Neonantal and Reproductive Health Studies<br>Stock, Sarah; The University of Edinburgh Usher Institute of Population Health Sciences and Informatics<br>Reynolds, Rebecca; The University of Edinburgh Centre for Cardiovascular Science, Queen's Medical Research Institute<br>Norrie, John; The University of Edinburgh Usher Institute of Population Health Sciences and Informatics, Edinburgh Clinical Trials Unit<br>Makwakwa, Enita; Malawi Epidemiology and Intervention Research Unit<br>Freyne, Bridget; University of Liverpool Institute of Infection and Global Health; University of Malawi College of Medicine, Paediatric Department<br>Campbell, Harry; The University of Edinburgh Usher Institute of Population Health Sciences and Informatics, Centre for Global Health Research<br>Norman, Jane; University of Bristol, Faculty of Health Sciences<br>Wood, Rachael; NHS National Services Scotland, Information Services Division; University of Edinburgh Salvesen Mindroom Research Centre
**Primary Subject Heading**:	Health services research
Secondary Subject Heading:	Global health, Obstetrics and gynaecology, Paediatrics, Health informatics
Keywords:	Information management < BIOTECHNOLOGY & BIOINFORMATICS, International health services < HEALTH SERVICES ADMINISTRATION & MANAGEMENT, Quality in health care < HEALTH SERVICES ADMINISTRATION & MANAGEMENT, NEONATOLOGY, OBSTETRICS

SCHOLARONE™
Manuscripts

I, the Submitting Author has the right to grant and does grant on behalf of all authors of the Work (as defined in the below author licence), an exclusive licence and/or a non-exclusive licence for contributions from authors who are: i) UK Crown employees; ii) where BMJ has agreed a CC-BY licence shall apply, and/or iii) in accordance with the terms applicable for US Federal Government officers or employees acting as part of their official duties; on a worldwide, perpetual, irrevocable, royalty-free basis to BMJ Publishing Group Ltd ("BMJ") its licensees and where the relevant Journal is co-owned by BMJ to the co-owners of the Journal, to publish the Work in this journal and any other BMJ products and to exploit all rights, as set out in our licence.

The Submitting Author accepts and understands that any supply made under these terms is made by BMJ to the Submitting Author unless you are acting as an employee on behalf of your employer or a postgraduate student of an affiliated institution which is paying any applicable article publishing charge ("APC") for Open Access articles. Where the Submitting Author wishes to make the Work available on an Open Access basis (and intends to pay the relevant APC), the terms of reuse of such Open Access shall be governed by a Creative Commons licence – details of these licences and which Creative Commons licence will apply to this Work are set out in our licence referred to above.

Other than as permitted in any relevant BMJ Author's Self Archiving Policies, I confirm this Work has not been accepted for publication elsewhere, is not being considered for publication elsewhere and does not duplicate material already published. I confirm all authors consent to publication of this Work and authorise the granting of this licence.

For peer review only

Informing prevention of stillbirth and preterm birth in Malawi: development of a minimum dataset for health facilities participating in the DIPLOMATIC collaboration

Word count: 4119

List of authors:

Beatrix von Wissmann, PhD, NHS National Services Scotland, Information Services Division, Gyle Square, 1 South Gyle Crescent, Edinburgh EH12 9EB, UK, beatrix.vonwissmann@nhs.net, +44 141 2014917 (Corresponding Author)

Elizabeth Wastnedge, MB ChB, MRC Centre for Reproductive Health, The University of Edinburgh, Edinburgh, UK

Donald Waters, MB ChB, MRC Centre for Reproductive Health, The University of Edinburgh, Edinburgh, UK

Luis A Gadama, Department of Obstetrics and Gynaecology, University of Malawi, College of Medicine, Blantyre, Malawi

Queen Dube, Paediatric Department, College of Medicine, University of Malawi, Blantyre, Malawi

Clemens Masesa, Malawi-Liverpool-Wellcome Trust Clinical Research Programme, Blantyre, Malawi

Elizabeth Chodzaza, PhD, RNM, Faculty of Midwifery, Neonantal and Reproductive Health Studies, Kamuzu College of Nursing, University of Malawi, Blantyre, Malawi

Sarah J Stock, PhD, Usher Institute of Population Health Sciences and Informatics, University of Edinburgh, Edinburgh, UK

Rebecca M Reynolds, Prof, PhD, Centre for Cardiovascular Science, University of Edinburgh, Queen's Medical Research Institute, Edinburgh, UK

John Norrie, MSc, Edinburgh Clinical Trials Unit, Usher Institute of Population Health Sciences & Informatics, Edinburgh, UK

Enita Makwakwa, MPH, Malawi Epidemiology and Intervention Research Unit, Lilongwe, Malawi

Bridget Freyne, MB BCh BAO, Institute of Infection & Global Health, University of Liverpool, Liverpool, UK and University of Malawi College of Medicine, Blantyre, Malawi

Harry Campbell, Prof, Centre for Global Health Research, Usher Institute of Population Health Sciences and Informatics, Medical School, University of Edinburgh, Edinburgh, UK

Jane E Norman, Prof, MD, Faculty of Health Sciences, University of Bristol, Bristol, UK

Rachael Wood, PhD, NHS National Services Scotland, Information Services Division, Edinburgh, UK and Salvesen Mindroom Research Centre, University of Edinburgh, Edinburgh, UK

For peer review only

For peer review only - http://bmjopen.bmj.com/site/about/guidelines.xhtml

On behalf of the DIPLOMATIC collaboration (Catherine Bamuya, Esmie Banda, James Boardman, Effie Chipeta, Mia Crampin, Sarah Cunningham Burley, Jean Desire Kabamba, Elizabeth Grant, Caroline Hollins Martin, Aisha Holloway, Khondwhani Kawaza, Doris Kayambo, Corrine Love, Brian Magowan, Monica Malata, Martha Masamba, Fadhila Mazanderani, Gladys Membe- Gadama, Catherine Mkandawire, Patricia Munthali, Peter Mwaba, Shakira Namisengo, Everist Njelesani, Linda Nyondo-Mipando, Hilary Pinnock, Muriel Syacumpi, Frank Taulo, Alexandra Viner)

For peer review only

For peer review only - http://bmjopen.bmj.com/site/about/guidelines.xhtml

ABSTRACT

Objective

The global research group, DIPLOMATIC (Using eviDence, Implementation science, and a clinical trial PLatform to Optimise MATernal and newborn health in low Income Countries), aims to reduce stillbirths and preterm births and optimise outcomes for babies born preterm. Minimum datasets for routine data collection in healthcare facilities participating in DIPLOMATIC (initially in Malawi) were designed, to assist understanding of baseline maternal and neonatal care processes and outcomes, and facilitate evaluation of improvement interventions and pragmatic clinical trials.**Design**

Published and grey literature was reviewed alongside extensive in-country consultation to define relevant clinical best practice guidance, and the existing local data and reporting infrastructure, to identify requirements for the minimum datasets. Data elements were subjected to iterative rounds of consultation with topic experts in Malawi and Scotland, the relevant Malawian professional bodies, and the Ministry of Health in Malawi to ensure relevance, validity, and feasibility.

Setting

Antenatal, maternity and specialist neonatal care in Malawi.

Results

The resulting three minimum datasets cover the maternal and neonatal healthcare journey for antenatal, maternity and specialist neonatal care, with provision for effective linkage of records for mother/baby pairs. They can facilitate consistent, precise recording of relevant outcomes (stillbirths, preterm births, neonatal deaths), risk factors and key care processes.

Conclusions

Poor quality routine data on care processes and outcomes constrains healthcare system improvement. The datasets developed for implementation in DIPLOMATIC partner facilities reflect, and hence support delivery of, internationally agreed best practice for maternal and newborn care in low income settings. Informed by extensive consultation, they are designed to integrate with existing local data infrastructure and reporting as well as meeting research data needs. This work provides a transferable example of strengthening data infrastructure to underpin a learning healthcare system approach in low income settings.

DIPLOMATIC is funded by the UK National Institute for Health Research.

For peer review only

For peer review only - http://bmjopen.bmj.com/site/about/guidelines.xhtml

STRENGTH AND LIMITATIONS OF THIS STUDY

- In line with recommended practice, the datasets were designed using a multimodal approach, combining an in depth review of the contextual information and published and grey literature with expert input.
- Delphi methods to gain expert consensus were considered, but deemed unsuitable due to the large number of variables, many of which form interconnected constructs and could thus not be changed independently of each other.
- Instead of a Delphi survey, iterative rounds of consultation with DIPLOMATIC collaborators and other stakeholders in Malawi were undertaken, which were essential to facilitate Malawian ownership, increasing the likelihood that study recommendations can be implemented and scaled up to increase impact.
- One limitation of this inclusive approach is that the datasets are more extensive than other exemplars of national minimum datasets.
- However the approach ensures that the datasets can integrate with existing systems and meet local data requirements, and also have the capacity to evaluate other Ministry of Health initiatives, rather than addressing research requirements only.

For peer review only

For peer review only - http://bmjopen.bmj.com/site/about/guidelines.xhtml

INTRODUCTION

The global burden of stillbirths and preterm births remains significant. According to recent global estimates, 2.6 million babies were stillborn at 28 completed weeks gestation or over in 2015.[1] Half of stillborn babies died during labour.[1] Complications of preterm birth (defined as delivery before 37 completed weeks gestation) accounted for nearly one fifth of deaths in under five year old children globally (18%) in 2016.[2] Babies born preterm, who survive, remain at risk of developmental delay,[3] and long term physical morbidities.[4]

DIPLOMATIC

DIPLOMATIC (Using evi**D**ence, **I**mplementation science, and a clinical trial **PL**atform to **O**ptimise **MAT**ernal and newborn health in low **I**ncome **C**ountries) is a National Institute of Health Research (NIHR) funded global research group with partners in Malawi, Zambia and the United Kingdom (UK), which aims to reduce stillbirth and preterm birth, and optimise outcomes for babies born preterm. In Malawi and Zambia, an estimated 14,000 and 13,000 stillbirths occur every year (21.8 and 20.9 stillbirths per 1000 total births respectively).[1] An estimated 68,000 and 76,000 babies per year are born preterm, accounting for 10.5% of births in Malawi and 12% in Zambia.[5] Ranked by increasing rates, Malawi and Zambia were 160th and 155th out of 194 countries for rates of stillbirth in 2015 and 123rd and 127th out of 183 countries for rates of preterm birth in 2014, respectively.[1, 5] DIPLOMATIC aims to develop pragmatic clinical trials to test the effectiveness of evidence based practices and how best to implement them in low income settings. Consistent and accurate data are required from participating healthcare facilities to efficiently monitor intervention implementation and outcomes and hence trial results.

Establishing parallel data collection for research projects, rather than integrating this with routine data collection, can lead to a disjointed approach which risks duplication of effort and waste of scarce resources. A learning health system approach has been identified in similar low income settings, as an effective means of combining data intelligence for quality improvement, with research on the implementation of new interventions and optimising their effectiveness. [6, 7] In this context, the learning health system was characterised by a strong stakeholder network, and facilitation of local application of data intelligence, to allow faster integration of evidence based interventions, and efficient use of the same data to drive research. [7]

For peer review only - http://bmjopen.bmj.com/site/about/guidelines.xhtml

Current perinatal data infrastructure in Malawi

To ensure that the data collection for DIPLOMATIC would integrate with and strengthen existing systems, the collaboration undertook a baseline, in-depth review of the existing data infrastructure relating to perinatal health in Malawi.

Vital events registration in Malawi is mandated by the National Registration Act of 2010, but coverage of birth and death registration is still incomplete, though efforts to improve coverage are in progress.[8, 9]

Individuals hold their own medical records as ‘health passports’ (structured paper based records), which they present whenever accessing healthcare. For women of childbearing age, the health passport records a summary of medical and obstetric history, contraceptives, antenatal care (ANC), and the delivery record. Neonates receive their own passport at birth. Most district and central hospitals maintain paper based patient notes when providing inpatient perinatal care. For specialist neonatal care, a standardised admission form is available. A brief note is made in the patients’ health passport at discharge,[10] which is usually the main source of information to clinicians on patients’ history in case of readmission, as retrieval of patient notes held by healthcare facilities is difficult. Neonatal and maternal records are not routinely linked, and if the mother is not present or deceased, her information is not available for the care of the neonate. An electronic patient record system for ANC is available, but uptake is limited to date (24 out of 85 hospitals and 12 out of 542 health centres across Malawi, as of May 2019).[11] An application for immediate electronic data capture on neonatal admissions (NeoTree) was developed recently and tested in one hospital.[12] This application is designed as a decision support tool, to improve quality of care, and does not have any reporting functionality for integration with local data systems as yet.[12]

In addition to individual patient notes, healthcare facilities maintain ‘registers’ (individual level paper logs) for clinical care including ANC, maternity/birth record, neonatal resuscitation, and Kangaroo Mother Care (KMC), with partial overlap in data items recorded. Aggregate data on healthcare activity are regularly extracted from registers and reported to the Ministry of Health (MoH), via the District Health Information System.[10]

Importance of high quality data

Stillbirths, and infant morbidity and mortality associated with preterm births are markers of maternal health and markers of access to high quality care in pregnancy, especially around the time of childbirth.[13, 14] However, global and national estimates of stillbirths and premature births are

For peer review only - http://bmjopen.bmj.com/site/about/guidelines.xhtml

frequently constrained by data quality.[1, 5, 15] High quality data are required to measure the burden of stillbirths and premature births, to assess effectiveness of interventions, and to monitor the benefits of investment to implement evidence based interventions (or the adverse consequences of a lack of investment). The importance of improving medical records and healthcare information systems, in order to allow monitoring and evaluation, and to improve performance and ultimately outcomes, has also been highlighted as the second of eight WHO standards for improving the quality of maternal and newborn care in health facilities.[16]

Aim of this work

The aim of this work was to design a minimum dataset, which can be piloted and implemented across all healthcare facilities participating in DIPLOMATIC, to enable electronic collection of consistent and accurate data on perinatal outcomes, risk factors and the coverage of key care processes and treatments. Whilst the DIPLOMATIC collaboration spans both Zambia and Malawi, this initial work focused on Malawi, with a view to transferring the equivalent data collection, adapted to the Zambian context, in a second stage.

For peer review only

METHODS

The minimum datasets were drawn up using a multimodal approach, building on review of the literature and existing data infrastructure for identification of data requirements, followed by consultation with project collaborators and wider local stakeholders in Malawi.[17, 18]

Identification of data requirements

Health passports, neonatal admission forms, and standard registers and the corresponding aggregate data returns to the MoH were reviewed. Variables required for the standard registers and to fulfil aggregate reporting requirements to the MoH were identified for inclusion in the datasets. The resulting list of variables was compared to relevant WHO guidance on ANC,[19] maternal health ,[20] and newborn health,[21] to ensure that the proposed datasets could be used to assess coverage of recommended clinical best practice. Finally, agreed quality indicators for maternity and neonatal services,[16, 22, 23] and existing minimum datasets,[24, 25] were reviewed to identify additional relevant variables. Wherever possible, international norms and standards were used to underpin definitions.[26]

For peer review only - http://bmjopen.bmj.com/site/about/guidelines.xhtml

Consultation

Identification of the data requirements was followed by iterative rounds of consultation to refine the datasets based on critical evaluation by DIPLOMATIC collaborators and wider stakeholders in Malawi, who provided topic expertise and insights on Malawian health data structure and management:

DIPLOMATIC collaborators were consulted on the appropriateness and feasibility of the draft datasets. In addition to requesting electronic feedback from all collaborators, phone, skype and e-mail conversations with relevant Malawi based experts amongst the collaborators ensured detailed input from obstetricians, paediatricians, midwives, epidemiologists and data scientists. Feedback was recorded against the relevant variables. Where conflicting comments were received, alignment with existing data infrastructure was prioritised, to ensure the data would meet local requirements. The datasets were updated accordingly and shared again with collaborators and discussed at the DIPLOMATIC management group. The draft datasets were shared with relevant professional bodies in Malawi (Nurses and Midwives Council, Association of Obstetricians and Gynaecologists, Association of Paediatricians) facilitated by DIPLOMATIC collaborators who are members of the respective bodies. The draft datasets were also shared with colleagues from the MoH Directorate of Reproductive Health and Directorate of Quality Management and Digital Health. Finally, the datasets were presented to the Safe Motherhood Technical Working Group, for endorsement by the MoH. The Technical Working Group has multi-sectoral membership including representation from the MoH and the health sector as well as the third sector and academia, and acts as a platform for policy dialogue and coordination of work.

Patient and Public involvement

Patients were not involved in the development of the datasets, but as set out in the DIPLOMATIC stakeholder engagement plan, the extensive experience of local host institutions for the DIPLOMATIC project (University of Malawi College of Medicine; Malawi Liverpool Wellcome Trust, Malawi Epidemiology and Intervention Research Unit) will be used to develop a program for public awareness of the interventions to be implemented (and to be evaluated using the datasets as described here), which will be supported by the Science Communication team at Malawi Liverpool Wellcome Trust. This will involve consultation with Community Groups, Women's Groups, Patients and Healthcare Workers and will involve multimedia platforms and local meetings.

For peer review only - http://bmjopen.bmj.com/site/about/guidelines.xhtml

RESULTS

Minimum dataset

Three minimum datasets were drafted to cover ANC, inpatient maternity care (including delivery and care of neonates in maternity settings, and care for miscarriages or complications of miscarriages), and specialist neonatal care. Figure 1 provides a flowchart summarising the datasets design process. Personal identifiers and demographic variables were included in each of the three datasets to ensure individuals can be uniquely identified and their records linked for care across different settings and at different facilities (horizontal linkage for the same individual during a single pregnancy and subsequent pregnancies, and vertical linkage for mothers and their babies). Malawi is in the process of implementing a unique health identification (UHID) system. Generation of a UHID will be facilitated through registration at the point of care, based on verification of identity using the recently introduced national ID (or birth certification). Use of a Quick Response (QR) code on health records for ease and speed of identification of the patient is being considered by the MoH. Each of the three DIPLOMATIC datasets allows recording of the local health record identifier, and this will be used to record the UHID as it embeds into health care record use in Malawi. Consistent use of the UHID across records of care will greatly support data linkage and may eventually allow automated imports of demographic details into the patient record, thus reducing data entry effort. However experience from Scotland shows that linkage of maternal and neonatal health records can remaining challenging, even when a unique health identifier is well established. The demographic and ID section of each of the three DIPLOMATIC datasets, is designed to facilitate data linkage, even in the absence of or in the event of a failure of linkage through a UHID.

Figure 1: Flowchart summarising the datasets design process [submitted as separate file]

To mitigate the risk of incomplete information due to patients receiving some elements of their care in facilities not participating in DIPLOMATIC, the datasets were designed for a hybrid approach between contemporaneous and retrospective data collection. Data would ideally be collected in each of the settings as care is completed (ANC and maternity and neonatal inpatient). However, the datasets also provide the option to capture retrospective summary information if the relevant data was not captured at the time the care was delivered. The maternity dataset thus includes a summary section on ANC, and the specialist neonatal care dataset allows retrospective recording of summary

For peer review only - http://bmjopen.bmj.com/site/about/guidelines.xhtml
For peer review only

of maternity/delivery care, if required (Figure 2). If the relevant data is captured electronically at the point of care, these retrospective summary sections can be auto-populated.

Figure 2: DIPLOMATIC datasets structure [submitted as separate file] (horizontal lines between the three ID/demography elements highlight that these facilitate the linkage of the different datasets for the same individual and/or mother baby pairings; different shading within the maternal discharge and the specialist neonatal care datasets show the retrospective summary sections).

The three datasets (summarised in Tables 1-3, available in full on Open Science Framework[17]) included variables to record:

- the main outcomes of interest to the collaboration (stillbirths, preterm births and neonatal deaths), including variables to record estimated date of delivery (EDD), and pregnancy outcome, and ICD10 coding for cause of death for stillbirths and neonatal deaths where available.
- key clinical data on potential risk factors and mediators for these outcomes (factors on the causal pathway), including the required variables on obstetric history to derive the Robson criteria (which classify women undergoing caesarean sections into groups defined by a global standard, for comparison of caesarean rates between and within healthcare facilities), underlying maternal conditions of relevance to the current pregnancy, and neonatal observations and underlying conditions.
- process measures for key aspects of routine healthcare as potential modifiers for these outcomes (factors increasing/ reducing risk of adverse outcomes), for ANC, delivery and immediate care of the newborn (including emergency interventions), and specialist neonatal care.

For peer review only - http://bmjopen.bmj.com/site/about/guidelines.xhtml

Table 1: Antenatal care (ANC) dataset structure and summary of included variables

when recorded	group	variable	comment
Booking contact* (cross check ID at each contact)	unique ID	maternal unique ID	record linkage
	ID& demography	maternal names (incl parents' names), date of birth, district & neighbourhood<br>maternal education<br>maternal local ID & local file numbers	record linkage failsafe (building on experience of requirements to uniquely identify individuals from Karonga HDSS**)<br>measure of socioeconomic status<br>cross-reference local files
All ANC contacts	ANC episode details	facility, level of ANC and care provider	level of care
	contact date	contact date	
	observations & tests	fetal heart rate, symphysis fundal height, any ultrasound scan (USS), maternal haemoglobin, blood pressure, urine protein, infections (syphilis, HIV and Hep B status)	for any USS after booking reason is recorded, many of these observations & tests are required for mandatory reporting to MoH
	care & treatment	tetanus toxoid vaccine doses, malaria prophylaxis, iron and folic acid tablets, preventive anthelmintic,<br>For HIV positive women: drugs for prevention of mother to child transmission (PMTCT)	many of these process measures are required for mandatory reporting to MoH
	high risk pregnancy indicator	indicator of whether woman meets any of the WHO high risk criteria	
Booking contact	EDD (and method of estimation)	EDD; USS before or at booking; LMP	EDD is recorded at booking alongside methods used for the estimate (USS, LMP, symphysis fundal height), EDD can subsequently only be altered once - and only on the basis of additional information provided by a first USS occurring after the booking visit
	obstetric, medical & behavioural history	obstetric history (previous pregnancies, deliveries and c-sections)<br>medical history (weight, height, blood group, HIV status, tetanus vaccination history)<br>behaviour (smoking, alcohol, drugs)	allows derivation of Robson criteria

For peer review only - http://bmjopen.bmj.com/site/about/guidelines.xhtml

*Booking contact: First (booking) visit as captured in the ANC Register, at which a full history is taken and, initial screening for medical, psychological and social risk factors takes places. Estimated date of delivery will also be recorded at the booking visit, although this may subsequently be amended if the first ultrasound scan for the pregnancy takes place after the booking visit.

**Karonga Health and Demographic Health Surveillance System (HDSS)[27]

Abbreviations used in table 1: Antenatal care (ANC), caesarean section (c-section), estimated date of delivery (EDD), human immunodeficiency virus (HIV), Hepatitis B (Hep B), identifier (ID), International Classification of Diseases version 10 (ICD10), last menstrual period (LMP), Ministry of Health (MoH), prevention of mother to child transmission (PMTCT), ultrasound scan (USS), World Health Organization (WHO)

For peer review only - http://bmjopen.bmj.com/site/about/guidelines.xhtml

Table 2: Maternal discharge dataset (including delivery record) structure and summary of included variables

when recorded	group	variable	comments
All discharges	maternal unique ID	maternal unique ID	record linkage
	ID& demography	maternal names (incl parents' names), date of birth, district & neighbourhood	record linkage failsafe (building on experience of requirements to uniquely identify individuals from Karonga HDSS*)
		maternal education	measure of socioeconomic status
		maternal local ID & local file numbers	cross-reference local files
All discharges	episode dates & details	admission and discharge dates, facility, admission from, discharge to<br>delivery and miscarriage episodes only: care provider staff group	
	condition & outcome	reason for admission (category and cause coded in ICD10), condition on discharge	condition on discharge indicates the outcome i.e. miscarriage, delivered or is still pregnant at the end of the care episode
Delivery and miscarriage episodes	ANC summary	obstetric, medical & behavioural history summary	to be autofilled if ANC took place in DIPLOMATIC participating facility, to be manually recorded retrospectively if ANC took place in facility not participating in DIPLOMATIC
		date of booking contact**	
		care & treatment summary	
		high risk pregnancy indicator, multiple pregnancy	
		EDD (and methods of estimation) summary	
Delivery record mother	outcome & details	number of babies, outcome of pregnancy, birth order, date, time, place, presentation, mode, indications for assisted delivery,	
	observation & tests	partograph used; HIV test, duration of stages of labour,	many of these observations & tests are required for mandatory reporting to MoH
	care & treatments	<u>preventive / routine:</u> antenatal steroids, magnesium sulfate, labour induction, augmentation of labour, analgesia, uterotonic for active management third stage of labour, episiotomy, companion present;<br><u>emergency:</u> uterotonic, anticonvulsive, antibiotic, blood transfusion, manual removal of placenta	many of these process measures are required for mandatory reporting to MoH

For peer review only - http://bmjopen.bmj.com/site/about/guidelines.xhtml

	discharge record	iron and folic acid tablets, preventive anthelminitic, contraception	
Delivery record baby	unique ID	baby unique ID	
	observations	all: sex, weight; livebirths: Apgar score, crown-heel length, head circumference	
	care & treatment	routine: dried & wrapped, delayed cord clamping, cord cut with sterile blade, skin to skin contact initiated, breastfeeding initiated, Vitamin K given, tetracycline eye ointment, BCG & OPV vaccine, PMTCT initiated (if mother HIV positive); emergency/supportive: resuscitation at birth, systemic antibiotics, kangaroo mother care (KMC) details, other thermal support	many of these process measures are required for mandatory reporting to MoH
	condition	congenital malformations, other perinatal complications	
	discharge record	date, feeding at discharge (mode & content), on KMC at discharge, discharge provider	
Miscarriages	miscarriage type & management	miscarriage type & management	
Stillbirths & Neonatal deaths	cause of death	immediate & underlying cause of death (coded in ICD10)	

*Karonga Health and Demographic Health Surveillance System (HDSS)[27]

**Booking contact: First (booking) visit as captured in the ANC Register, at which a full history is taken and, initial screening for medical, psychological and social risk factors takes places. Estimated date of delivery will also be recorded at the booking visit, although this may subsequently be amended if the first ultrasound scan for the pregnancy takes place after the booking visit.

For peer review only

For peer review only - http://bmjopen.bmj.com/site/about/guidelines.xhtml

Abbreviations used in table 2: Antenatal care (ANC), Bacillus Calmette–Guérin tuberculosis vaccine (BCG), identifier (ID), estimated date of delivery (EDD), International Classification of Diseases version 10 (ICD10), kangaroo mother care (KMC), Ministry of Health (MoH), Oral Polio Vaccine (OPV), prevention of mother to child transmission (PMTCT)

For peer review only

For peer review only - http://bmjopen.bmj.com/site/about/guidelines.xhtml

Table 3: Specialist neonatal care dataset structure and summary of included variables

when recorded	group	variable	use
All discharges	baby unique ID	baby unique ID	record linkage
	baby ID& demography	baby names, date of birth, sex, district & neighbourhood<br>baby local ID & local file numbers	record linkage failsafe<br>cross-reference local files
	maternal unique ID	maternal unique ID	record linkage
	maternal ID& demography	maternal names (incl parents' names), date of birth, district & neighbourhood<br>maternal education<br>maternal local ID & local file numbers	record linkage failsafe (building on experience of requirements to uniquely identify individuals from Karonga HDSS*)<br>measure of socioeconomic status<br>cross-reference local files
All discharges	episode dates & details	admission and discharge dates, facility, admission from, discharge to	
	condition	reason for admission (category and cause coded in ICD10),	
	observations	temperature & weight at admission	
	care & treatment	<u>emergency/supportive:</u> major invasive or therapeutic procedures, kangaroo mother care (KMC) details, other thermal support, respiratory support	many of these observations & tests are required for mandatory reporting to MoH
	discharge record	date, weight at discharge, feeding at discharge (mode & content), on KMC at discharge, discharge provider	
All discharges	Delivery record mother summary	EDD<br>outcome & details<br>observation & tests<br>care & treatments	to be autofilled if delivery took place in DIPLOMATIC participating facility, to be manually recorded retrospectively if delivery took place in facility not participating in DIPLOMATIC
	Delivery record baby summary	observations<br>care & treatments	
Neonatal deaths	cause of death	immediate & underlying cause of death (coded in ICD10)	

For peer review only - http://bmjopen.bmj.com/site/about/guidelines.xhtml

*Karonga Health and Demographic Health Surveillance System (HDSS)[27]

Abbreviations used in table 3: Antenatal care (ANC), identifier (ID), estimated date of delivery (EDD), International Classification of Diseases version 10 (ICD10), kangaroo mother care (KMC), Ministry of Health (MoH), prevention of mother to child transmission (PMTCT)

For peer review only

For peer review only - http://bmjopen.bmj.com/site/about/guidelines.xhtml

All variables and response options were supported by clear definitions, using internationally recognised norms where possible.[26] Validation cross-checks at data entry were built in to support data quality by design. The datasets were designed to include all required variables to replicate the standard registers used in the respective settings [ANC , maternity, helping babies breathe (neonatal resuscitation), KMC] and to allow generation of the respective mandatory reports for the MoH via the District Health Information System .

Consultation with the DIPLOMATIC collaborators, and wider stakeholders and professional bodies in Malawi resulted in refinement of a number of variables, including additional personal identifiers to create a failsafe for record linkage (based on local experience[27]), and the combination of variables that allow recording of the EDD and the method of estimation. Consultation input also ensured that the response options for all process measures were appropriate for the Malawian healthcare context, including response options to record that items were out of stock. The datasets were endorsed by the MoH through approval by the Safe Motherhood Technical Working Group.

DISCUSSION

The DIPLOMATIC minimum datasets were designed to cover the maternal and neonatal healthcare journey from ANC to specialist neonatal care. The datasets encompass the required elements to allow precise recording of the relevant outcomes (stillbirths, preterm births and neonatal deaths), as well as risk factors and key care processes. The datasets are aligned with international standards and definitions to ensure comparability of the results, and will enable DIPLOMATIC and the MoH to evaluate implementation and effectiveness of interventions (including care in line with existing policies). They were designed to integrate with and strengthen local data systems and meet reporting requirements, and to meet data needs to support decision making and learning for quality improvement in line with the WHO standards for improving the quality of maternal and newborn care in health facilities.[16] Consultation and refinement will need to continue during pilots in the DIPLOMATIC partner facilities, planned to commence in autumn 2020, to ensure the datasets remain aligned with MoH reporting requirements and to enable synergies with projects in the fast moving field of electronic medical record (EMR) developments, such as an eRegisters platform currently being piloted. In recent discussions the Quality Management and Digital Health (QMDH) Directorate under Malawi's MoH have provided their support to integrate the DIPLOMATIC minimum datasets into this eRegister platform.

For peer review only - http://bmjopen.bmj.com/site/about/guidelines.xhtml

Data collection on preterm births and stillbirths is reliant on accurate information on gestation, which underpins the definitions of both concepts. WHO recommends the integration of an ultrasound scan before 24 weeks of gestation into routine ANC, as the most accurate method of determining gestation.[19] At present, ultrasound scans are not part of routine ANC in Malawi, but this was identified as a key evidence-based priority and one of the interventions to be implemented in the healthcare facilities participating in DIPLOMATIC.

The methods of assessment of gestational age not only determine the precision of estimates for individual pregnancies, but can also lead to systematic differences in population level estimates of prematurity rates,[5] (as exemplified by changes in reporting in the United States [28]). However, most data sources contributing to recent global, regional and national estimates of premature birth rates reported insufficient details on the methods to determine gestational age, to allow the estimates to take this parameter into account.[5] In contrast, the DIPLOMATIC datasets include the relevant parameters to allow recording of whether and when an ultrasound scan was carried out to date the pregnancy, or which other method were used (last menstrual period, symphysis fundal height), alone or in combination to estimate gestation. These datasets thus allow estimates of preterm birth rates to be adjusted for the methods used to estimate gestational age. The datasets will also allow other important factors to be taken into account, such as multiplicity, and onset of delivery (spontaneous or provider initiated) for analysing both live and total births,[15] and maternal risk factors such as body mass index (BMI) and hypertensive conditions.[29]

The datasets include process measures on different elements of routine care. These elements serve to meet local reporting requirements, and by recording uptake of evidence based antenatal, maternal and neonatal care as recommended by WHO,[19-21] they facilitate monitoring of improvements in quality of care. However, each of these data elements also include response options to reflect that specific care in line with the protocols could not be carried out for example due to stock out of the relevant drugs, in recognition that resource limitations pose important barriers to implementation of care in line with these guidelines. Malawi has adopted the 'Every Newborn Action Plan',[22,30] and is one of the countries participating in the 'Network for Improving Quality of Care for Maternal, Newborn and Child Health', which aims to halve newborn and maternal deaths, and intrapartum stillbirths in participating countries over five years.[31] The process measures which will be captured in the DIPLOMATIC datasets are closely aligned to the WHO framework for improving quality of maternal and newborn care in healthcare settings,[16] and will facilitate reporting against a large proportion of the stipulated quality measures. The datasets allow facility based reporting against 73% (8/11) of core indicators for the framework, and 88% (32/36) of

For peer review only - http://bmjopen.bmj.com/site/about/guidelines.xhtml

outcome indicators and 77% (44/57) of process/output indicators under the first strategic area of the framework which relates to evidence based practices for routine care and management of complications.

DIPLOMATIC is staying abreast of the changing electronic health records landscape in Malawi, and is proactively engaging with the Quality Management and Digital Health Directorate under the MoH which is taking the lead to guide the systems to be development and supported. The collaboration also benefits from the insight of experts, who successfully implemented an electronic data-collection tool in the tertiary care hospital in Blantyre (Surveillance Programme of IN-patients and Epidemiology –SPINE). [32] However, paper records are currently still an important part of recording and sharing health care data in Malawi, and the technical implementation of the DIPLOMATIC minimum datasets will take this into account. The DIPLOMATIC minimum datasets will ideally feed off structured patient records that include the variables of interest, but these may be paper based or electronic, using real-time or retrospective methods of data collection depending on system functionality. Engagement with staff in the participating sites, to discuss advantages of different ways of data capture, to optimise ease of data recording, and to establish how this can be supported through existing clerking staff, will be essential to address the challenges of consistent data recording, in the face of pressures on clinical staff time.

Supporting staff to facilitate optimal use of the data will be an important part of piloting and implementation of the datasets. Electronic data can significantly reduce the work required to extract data for audits and patient reviews (compared to extraction from paper records). Experiencing benefits from the data will enhance ownership and buy in from clinical staff, and will be a prerequisite to ensuring sustainability of the datasets. High quality data providing continuous feedback on quality and safety of care and patient outcomes to healthcare providers can underpin a ‘learning health system’, which allows data intelligence to foster learning and inform decisions.[33] Data linkage plays an important part in this when transfer of patients across facilities is common. Learning health system approaches have been shown to result in improved data and care quality in limited-resource settings similar to Malawi.[6,7] The DIPLOMATIC datasets have the potential to facilitate a learning health system approach.

Strengths and limitations

In line with recommended practice, the datasets were designed using a multimodal approach, combining an in depth review of the contextual information and published as well as grey literature with expert input.[34] Delphi methods to gain expert consensus were considered, but deemed

For peer review only - http://bmjopen.bmj.com/site/about/guidelines.xhtml

unsuitable due to the large number of variables, many of which form interconnected constructs and could thus not be changed independently of each other. Instead of a Delphi survey, iterative rounds of consultation with DIPLOMATIC collaborators and other stakeholders in Malawi were undertaken. Group discussions were helpful to find agreement on complex constructs, e.g. recording of EDD and methods used for its estimation. Whilst this process also took considerable time to complete, partly due to competing demands for the time of all of the relevant experts, it facilitated agreement on the variables to be prioritised for inclusion in the datasets. Sharing the draft datasets with professional associations and the relevant directorates of the MoH Malawi, and gaining approval by the Safe Motherhood Technical Working Group, was important to ensure there were no objections to the datasets, and to strengthen Malawian ownership, increasing the likelihood that any recommendations arising from future study findings can be implemented and scaled up to increase impact.

One limitation of the DIPLOMATIC datasets is that they are more extensive than other exemplars of national minimum datasets on maternal and neonatal health.[24, 25] This poses a challenge for implementation of these datasets in the context of stretched clinical staff, a background of predominantly paper based data recording and mixed success of electronic data systems.[10] However it was deemed essential to ensure that the datasets incorporate all data items required for standard registers and can fulfil the Malawian national reporting obligations to the MoH, so the datasets can be embedded in existing systems and strengthen these (in line with the 'record once, use often' principle). This approach avoided creation of parallel datasets specific only to the research data needs, which would have been smaller, but would have overlapped to a large extent with existing paper based registers, leading to duplication of effort and no benefit to the existing system.

The DIPLOMATIC collaboration is focussed on health care interventions, and the minimum datasets can only capture maternal and newborn care in health facilities. Home births and complications associated with these, or stillbirths out with health facilities, would only be capture if aftercare was received in a health facility, or if recorded as part of a subsequent health care episode.

CONCLUSIONS

By combining contextual work with expert input, it was possible to design datasets, which build on existing structures, and can support local reporting requirements, as well as meeting the research data needs of DIPLOMATIC. The work to design the datasets highlights the need for a collaborative approach to ensure synergies and to strengthen existing systems, and avoid duplication of data and effort. Consultation on data collection is also essential to ensure local ownership of the data and any

For peer review only

findings arising from it. The datasets have the potential to underpin a 'learning health system' approach, and their piloting and implementation will be aimed at supporting quality of care to improve outcomes. Implementation of these datasets could thus contribute to fulfilling the aims of the Network for Improving Quality of Care for Maternal, Newborn and Child Health, in particular to strengthening of the health information system, 'to enable early, appropriate action to improve the care of every woman and newborn' (Standard 2).[16] The datasets may also serve beyond the use by facilities participating in DIPLOMATIC, most immediately as an exemplar on how to incorporate methods of gestation estimates into routine data collection, in settings where access to early pregnancy ultrasound is limited and different methods are likely to be in use. In a wider context they also serve as a transferable example of strengthening data infrastructure and use of robust data, to underpin a learning healthcare system approach in low income settings.

DATA SHARING STATEMENT

The full specifications of the datasets, the development of which is described in this manuscript, are available on Open Science Framework: von Wissmann B, Gadama L, Dube Q, et al. The DIPLOMATIC Collaboration, Work Package 2: Core datasets. 2020. https://osf.io/d3s7j [17].

ACKNOWLEDGEMENTS

This work would not have been possible without consultation with stakeholders from the Ministry of Health Malawi, the Malawian Nurses and Midwives Council, the Malawian Association of Obstetricians and Gynaecologists, and the Malawian Association of Paediatricians.

We would like to thank Jakub Adamów for producing the graphic for this paper.

CONTRIBUTORS

BvW and RW designed the protocol for the development of the datasets and drafted the first version of the datasets. BvW wrote the first draft of the manuscript. BvW, EW, DW and RW synthesised the information needed to identify the dataset requirements. LAG, QD, CM, EC provided expert input on the dataset requirements, and current data infrastructure and management in Malawi. LAG, QD and EC led on engagement with the professional bodies. All authors (BvW, EW, DW, LAG, QD, CM, EC, SJS, RMR, JN, EM, BF, HC, JEN, RW) provided significant intellectual input to the iterative development of the datasets. All authors (BvW, EW, DW, LAG, QD, CM, EC, SJS, RMR, JN, EM, BF, HC, JEN, RW) provided input to drafting the manuscript.

For peer review only - http://bmjopen.bmj.com/site/about/guidelines.xhtml

Competing interests

SJS reports grants from NIHR HTA, non-financial support from HOLOGIC, non-financial support from PARSAGEN, non-financial support from MEDIX BIOCHEMICA, during the conduct of the study; and SJS declares being a member of the HTA General committee.

JN reports membership of the following NIHR boards: Commissioning Priority Review Decision-making committee (2015); Health Technology Assessment (HTA) Commissioning Board (2010-2016); HTA Commissioning Sub-Board (Expression of Interest) (2014); HTA Funding Boards Policy Group (2016-2019); HTA General Board (2016-2019); HTA Post-Board funding teleconference (2016-2019); NIHR Clinical Trials Unit Standing Advisory Committee (2018-present); NIHR HTA and Efficacy and Mechanism Evaluation Editorial Board (2014-2019); Pre-exposure Prophylaxis Impact Review Panel (2017).

JEN reports being named as Principal Investigator on government and charitable research grants to her institution which aim to improve pregnancy outcome. In the last three years she has provided consultancy to Pharma companies GSK and Dilafor: her institution was remunerated for this. Her institution has received travel and subsistence expenses from Merck to facilitate her speaking at a Merck sponsored symposium on metformin. She is on Subpanel A1 for REF, and on a Wellcome Trust Science interview panel, and receive personal renumeration for each.

FUNDING

This research was funded by the National Institute for Health Research (NIHR) (GHR Project: 17/63/08 DIPLOMATIC collaboration) using UK aid from the UK Government to support global health research. The views expressed in this publication are those of the authors and not necessarily those of the NIHR or the Department of Health and Social Care.

SJS is funded by a Wellcome Trust Clinical Career Development Fellowship (209560).

For peer review only

REFERENCES

1. Blencowe H, Cousens S, Jassir FB, et al. National, regional, and worldwide estimates of stillbirth rates in 2015, with trends from 2000: a systematic analysis. Lancet Glob Health. 2016;4(2):e98-e108. doi: 10.1016/S2214-109X(15)00275-2.

2. UN Inter-Agency Group for Child Mortality Estimation. Levels and trends in child mortality: Report 2017. New York; 2017. https://www.un.org/en/development/desa/population/publications/mortality/child-mortality-report-2017.asp (accessed 26/10/2019)

3. Bhutta AT, Cleves MA, Casey PH, et al. Cognitive and behavioral outcomes of school-aged children who were born preterm: a meta-analysis. JAMA. 2002;288(6):728-37.

4. Moster D, Lie RT, Markestad T. Long-term medical and social consequences of preterm birth. N Engl J Med. 2008;359(3):262-73. doi: 10.1056/NEJMoa0706475.

5. Chawanpaiboon S, Vogel JP, Moller AB, et al. Global, regional, and national estimates of levels of preterm birth in 2014: a systematic review and modelling analysis. Lancet Glob Health. 2019;7(1):e37-e46. doi: 10.1016/S2214-109X(18)30451-0.

6. Tuti T, Bitok M, Malla L, et al. Improving documentation of clinical care within a clinical information network: an essential initial step in efforts to understand and improve care in Kenyan hospitals. BMJ Glob Health. 2016;1(1):e000028. doi: 10.1136/bmjgh-2016-000028.

7. Irimu G, Ogero M, Mbevi G, et al. Approaching quality improvement at scale: a learning health system approach in Kenya. Arch Dis Child. 2018;103(11):1013-9. doi: 10.1136/archdischild-2017-314348.

8. Gadabu OJ, Ben-Smith A, Douglas GP, et al. Scaling up electronic village registers for measuring vital statistics in rural villages in Malawi. Public Health Action. 2018;8(2):79-84. doi: 10.5588/pha.17.0116.

9. Nichols EK, Giles D, Kang'oma S, et al. Rapid assessment of Malawi's civil registration and vital statistics system. Public health action. 2015;5(3):162-4. doi: 10.5588/pha.15.0021.

10. Tough AG, Lihoma P. Medical record keeping systems in Malawi: Is there a case for hybrid systems and intermediate technologies? Records Management Journal. 2018;28(3):265-77. doi.org/10.1108/RMJ-02-2018-0004

11. Baobab Health Trust. Where we work - Electronic medical record systems deployed in various health facilities nationwide. 2019 [27/05/2019]. http://baobabhealth.org/wherewework.php (accessed 27/05/2019).

12. Crehan C, Kesler E, Nambiar B, et al. The NeoTree application: developing an integrated mHealth solution to improve quality of newborn care and survival in a district hospital in Malawi. BMJ Glob Health. 2019;4(1):e000860. doi: 10.1136/bmjgh-2018-000860

13. Graham W, Woodd S, Byass P, et al. Diversity and divergence: the dynamic burden of poor maternal health. Lancet. 2016;388(10056):2164-75. doi: 10.1016/S0140-6736(16)31533-1. Epub 2016 Sep 16.

14. Lawn JE, Blencowe H, Waiswa P, et al. Stillbirths: rates, risk factors, and acceleration towards 2030. Lancet. 2016;387(10018):587-603. doi: 10.1016/S0140-6736(15)00837-5. Epub 2016 Jan 19.

15. Lee AC, Blencowe H, Lawn JE. Small babies, big numbers: global estimates of preterm birth. Lancet Glob Health. 2019;7(1):e2-e3. doi: 10.1016/S2214-109X(18)30484-4. Epub 2018 Oct 30.

For peer review only - http://bmjopen.bmj.com/site/about/guidelines.xhtml

16. World Health Organization. Standards for improving the quality of maternal and newborn care in health facilities. Geneva: WHO; 2016. https://www.who.int/maternal_child_adolescent/documents/improving-maternal-newborn-care-quality/en/ (accessed 26/10/2019).

17. von Wissmann B, Gadama L, Dube Q, et al. The DIPLOMATIC Collaboration, Work Package 2: Core datasets. 2020. https://osf.io/d3s7j (accessed 24/02/2020).

18. von Wissmann B, Wastnedge E, Waters D, et al. The DIPLOMATIC Collaboration, Work package 2: Contextual information. 2020. https://osf.io/dxwgy (accessed 24/02/2020).

19. World Health Organization. WHO recommendations on antenatal care for a positive pregnancy experience. Geneva: WHO; 2016. https://www.who.int/reproductivehealth/publications/maternal_perinatal_health/anc-positive-pregnancy-experience/en/ (accessed 26/10/2019).

20. World Health Organization. WHO Recommendations on maternal health. Geneva: WHO; 2017. https://www.who.int/maternal_child_adolescent/documents/maternal-health-recommendations/en/ (accessed 26/10/2019).

21. World Health Organization. WHO Recommendations on newborn health. Geneva: WHO; 2017. https://www.who.int/maternal_child_adolescent/documents/newborn-health-recommendations/en/ (accessed 26/10/2019).

22. World Health Organization. Every Newborn: an action plan to end preventable deaths. Geneva; 2014. https://www.who.int/maternal_child_adolescent/newborns/every-newborn/en/ (accessed 26/10/2019).

23. COMET initiative. Core Outcomes in Neonatology (COIN). 2018. http://www.comet-initiative.org/studies/details/842 (accessed 26/10/2019)

24. National Services Scotland Information Services Division. Scottish Morbidity Records 02 (SMR02) Maternity Inpatient and Day Case. http://www.ndc.scot.nhs.uk/Data-Dictionary/SMR-Datasets/SMR02-Maternity-Inpatient-and-Day-Case/ (accessed 26/10/2019).

25. National Services Scotland Information Services Division. Scottish Birth Records. http://www.ndc.scot.nhs.uk/National-Datasets/data.asp?SubID=2 (accessed 26/10/2019).

26. World Health Organization. WHO: recommended definitions, terminology and format for statistical tables related to the perinatal period and use of a new certificate for cause of perinatal deaths. Modifications recommended by FIGO as amended October 14, 1976. Acta Obstet Gynecol Scand. 1977;56(3):247-53.

27. Crampin AC, Dube A, Mboma S, Price,A. et al. Profile: the Karonga Health and Demographic Surveillance System. International journal of epidemiology. 2012;41(3):676-85. doi: 10.1093/ije/dys088. Epub 2012 Jun 22.

28. Martin JA, Osterman MJK, Kirmeyer SE, et al. Measuring gestational age in vital statistics data: transitioning to the obstetric estimate. National Vital Statistics Reports. 2015;64(5).

29. Goldenberg RL, Culhane JF, Iams JD, et al. Epidemiology and causes of preterm birth. Lancet. 2008;371(9606):75-84. doi: 10.1016/S0140-6736(08)60074-4.

30. Malawi Ministry of Health. Every Newborn Action Plan: An action plan to end preventable neonatal deaths in Malawi. Lilongwe: Malawi Ministry of Health; 2015. https://www.who.int/pmnch/media/events/2015/malawi_enap.pdf (accessed 26/10/2019).

For peer review only - http://bmjopen.bmj.com/site/about/guidelines.xhtml

31. World Health Organization. Quality, Equity, Dignity. A Network for Improving Quality of Care for Maternal, Newborn and Child Health. Geneva: WHO; 2017. https://www.who.int/maternal_child_adolescent/topics/quality-of-care/quality-of-care-brief-qed.pdf?ua=1 (accessed 26/10/2019).

32. SanJoaquin MA, Allain TJ, Molyneux ME, et al. Surveillance Programme of IN-patients and Epidemiology (SPINE): implementation of an electronic data collection tool within a large hospital in Malawi. PLoS Med. 2013;10(3):e1001400. doi: 10.1371/journal.pmed.1001400.

33. Budrionis A, Bellika JG. The Learning Healthcare System: Where are we now? A systematic review. J Biomed Inform. 2016;64:87-92. doi: 10.1016/j.jbi.2016.09.018. Epub 2016 Sep 28.

34. Svensson-Ranallo PA, Adam TJ, Sainfort F. A framework and standardized methodology for developing minimum clinical datasets. AMIA Jt Summits Transl Sci Proc. 2011;2011:54-8.

For peer review only

For peer review only - http://bmjopen.bmj.com/site/about/guidelines.xhtml

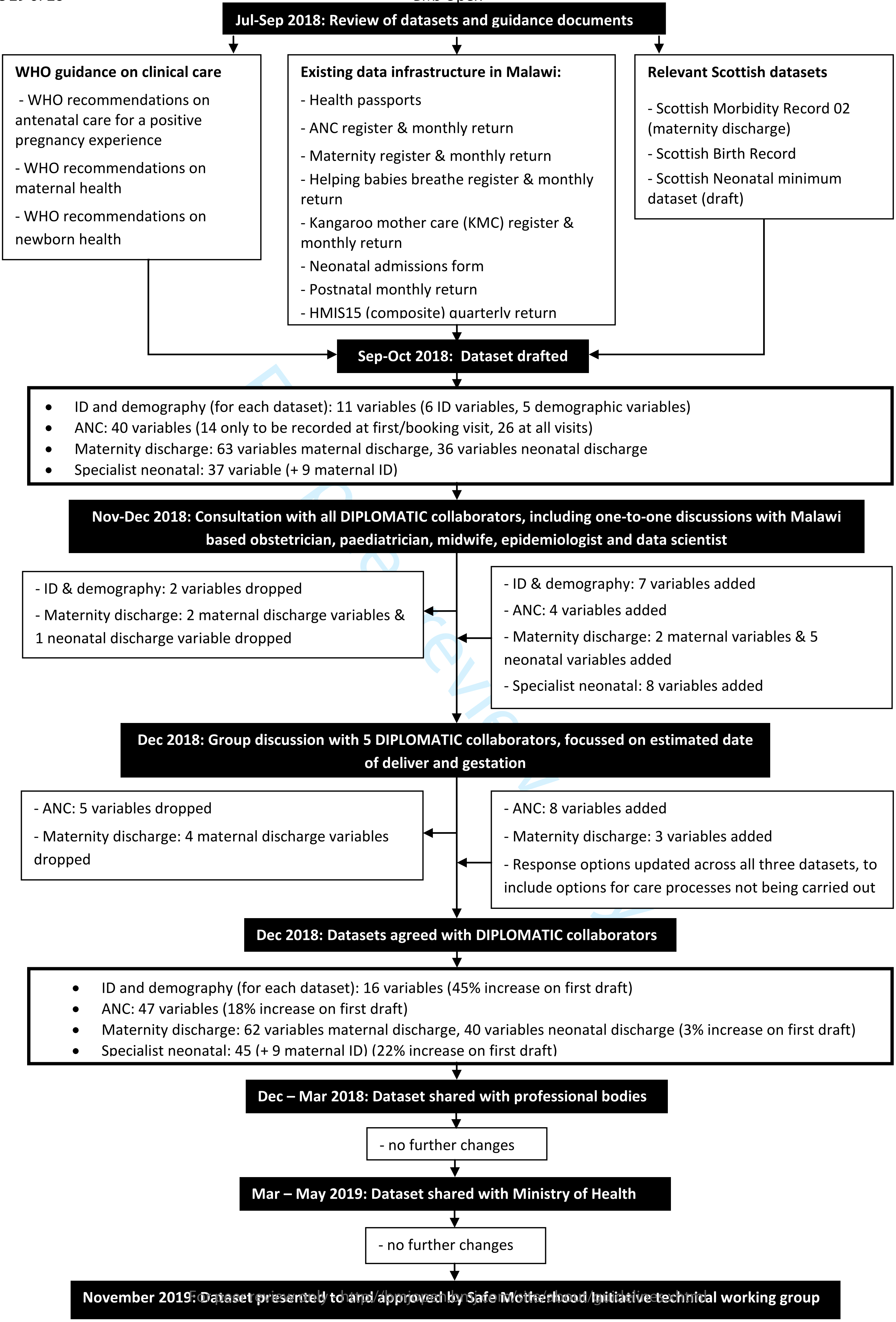

For peer review only - http://bmjopen.bmj.com/site/about/guidelines.xhtml

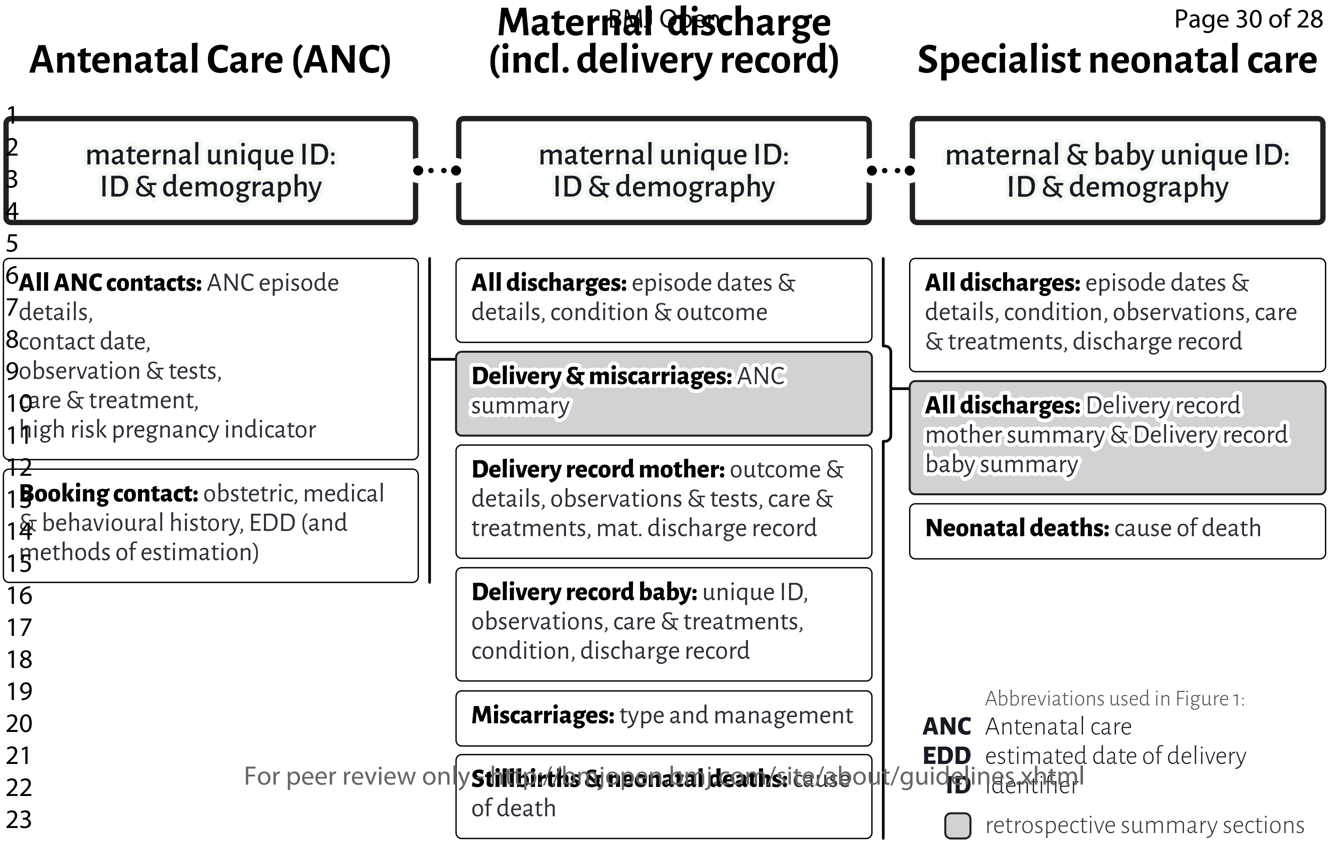

For peer review only - http://bmjopen.bmj.com/site/about/guidelines.xhtml
